# Supplementary material for: Supervised learning and model analysis with compositional data
Source: PLoS Comput Biol. 2023 Jun 30;19(6):e1011240. doi: 10.1371/journal.pcbi.1011240 (PMC10343141; doi:10.1371/journal.pcbi.1011240)
Supplement: S1 Appendix — Formal definitions of perturbations and estimators related to CFI and CPD. (PDF) [file pcbi.1011240.s001.pdf]

S1 Appendix: Details on CFI and CPD

A Perturbations

Formally, the multiplicative perturbation ψ and the fixed coordinate perturbation ϕ are defined as follows.

- For all $j \in \{1, \dots, p\}$, $x \in \mathbb{S}^{p-1}$ with $x^j \neq 1$ and $c \in [0, \infty)$, define

$$\psi_j(x, c) \coloneqq s_c(x^1, \cdots, x^{j-1}, cx^j, x^{j+1}, \cdots, x^p) \in \mathbb{S}^{p-1},$$

  where $s_c = 1/(\sum_{\ell \neq j}^p x^\ell + cx^j)$.

- For all $j \in \{1, \dots, p\}$, $x \in \mathbb{S}^{p-1}$ with $\sum_{\ell \neq j}^p x^\ell > 0$ and $c \in [0, 1]$, define the intervened composition by

$$\phi_j(x, c) \coloneqq (sx^1, \cdots, sx^{j-1}, c, sx^{j+1}, \cdots, sx^p) \in \mathbb{S}^{p-1},$$

  where $s = (1 - c)/(\sum_{\ell \neq j}^p x^\ell)$.

B Estimators

We propose to estimate CFI and CPD with the following two estimators.

- For i.i.d. observations $X_1, \dots, X_n$ and a differentiable function $f : \mathbb{S}^{p-1} \to \mathbb{R}$, we estimate the CFI for all $j \in \{1, \dots, p\}$ as

$$\hat{I}_f^j = \frac{1}{n} \sum_{i=1}^n \tfrac{d}{dc} f(\psi(X_i, c))\big|_{c=1}.$$

- For i.i.d. observations $X_1, \dots, X_n$ and a function function $f : \mathbb{S}^{p-1} \to \mathbb{R}$, we estimate the CPD for all $j \in \{1, \dots, p\}$ and $z \in [0, 1]$ as

$$\hat{S}_f^j(z) = \frac{1}{n} \sum_{i=1}^n f(\phi(X_i, z)) - \frac{1}{n} \sum_{i=1}^n f(X_i).$$
