# Supplementary material for: Supervised learning and model analysis with compositional data
Source: PLoS Comput Biol. 2023 Jun 30;19(6):e1011240. doi: 10.1371/journal.pcbi.1011240 (PMC10343141; doi:10.1371/journal.pcbi.1011240)
Supplement: S2 Appendix — Overview of different kernel types, details on how they connect to distances and description of weighted kernels. (PDF) [file pcbi.1011240.s002.pdf]

S2 Appendix: Details on kernels included in `KernelBiome`

A Overview of kernels in `KernelBiome`

In this section, we give additional details on the kernels used in `KernelBiome`. A full list of all kernels and their corresponding metrics together with a visualization on $\mathbb{S}^2$ is given in S7 Appendix.

As discussed in the main paper, we consider four types of kernels.

- **Euclidean** These are kernels that are used on Euclidean space but restricted to the simplex. This includes the *linear kernel* and the *RBF kernel*.

- **Probability distribution** These are kernels that are constructed from metrics between probability distributions. `KernelBiome` includes two parametric classes of kernels, the *Hilbertian kernel* and the *generalized-JS kernel*. These kernels correspond to multiple well-known metrics on probabilities such as the *chi-squared metric*, the *total-variation metric*, the *Hellinger metric* and the *Jensen-Shannon metric*.

- **Aitchison geometry** These are kernels that are constructed by using the centered log-ratio transform to project data on the simplex into Euclidean space and then combining it with a Euclidean kernel. `KernelBiome` includes the *Aitchison kernel* and the *Aitchison RBF kernel*. In order to allow for zeros, a small positive number c is added to each coordinate for all observations before applying the centered log-ratio transformation.

- **Riemannian manifold** These kernels are connected to the simplex via multinomial distributions and have been shown to empirically perform well on sparse text data mapped into the simplex. `KernelBiome` contains the *heat-diffusion kernels*.

For each type of kernel there are multiple parameter settings. Although users of the `KernelBiome` package can freely change the parameters, the default settings for `KernelBiome` for each type of kernel are provided by the package and are given in Table A.

Geometry	Kernel	Parameters	Number of kernels
Euclidean	linear	none	1
	RBF	$\sigma^2 \in \{10^{-2} \cdot m_1, 10^{-1} \cdot m_1, m_1, 10 \cdot m_1, 10^2 \cdot m_1, 10^3 \cdot m_1, 10^4 \cdot m_1\}$	7
Probability distributions	generalized-JS	$(a, b) \in \{(1, 0.5), (1, 1), (10, 0.5), (10, 1), (10, 10), (\infty, 0.5), (\infty, 1), (\infty, 10), (\infty, \infty)\}$	9
	Hilbertian	$(a, b) \in \{(1, -1), (1, -10), (1, -\infty), (10, -1), (10, -10), (10, -\infty), (\infty, -1), (\infty, -10)\}$	8
Aitchison geometry	Aitchison	$c \in \{\mu_X/2 \cdot 10^{-4}, \ldots, \min(\mu_X/2 \cdot 10^4, 10^{-2})\}$	9
	Aitchison-RBF	$c \in \{\mu_X/2 \cdot 10^{-4}, \ldots, \min(\mu_X/2 \cdot 10^4, 10^{-2})\}$, $\sigma \in \{c \cdot m_2 \cdot 10^{-1}, c \cdot m_2, c \cdot m_2 \cdot 10\}$	15
Riemannian manifold	heat-diffusion	$t = x^{\frac{2}{n-1}} \frac{1}{4\pi}$ for $x \in \{10^{-20}, \ldots, 10\}$	6

Table A. Default parameter grid in `KernelBiome`. m_1 and m_2 are the median heuristic for the RBF and Aitchison-RBF kernel, respectively, which depend on the data. μ_X is the minimal non-zero value in X. The zero grids for the Aitchison geometry kernels have an even logarithmic spacing and contain 9 and 5 parameters for the Aitchison and Aitchison-RBF, respectively. Similarly, the grid for x for the heat-diffusion kernel has an even logarithmic spacing with 6 values. There are a total of 55 kernels.

A.1 Connecting positive definite kernels to metrics

A semi-metric d satisfies all properties of a metric, except that $d(x, y) = 0$ does not imply $x = y$. This can happen because a kernel can map two different points in $\mathcal{X}$ to the same point in $\mathcal{H}_k$. Any fixed kernel k on $\mathcal{X}$ induces a semi-metric d_k on $\mathcal{X}$ defined for all $x, y \in \mathcal{X}$ by

$$d_k^2(x, y) = k(x, x) + k(y, y) - 2k(x, y). \tag{A}$$

This holds for all positive-definite kernels by Theorem 1.2 in S5 Appendix. In particular, this corresponds to the distance between the embedded points in the RKHS $\mathcal{H}_k$, that is,

$$\|k(x, \cdot) - k(y, \cdot)\|_{\mathcal{H}_k} = d_k(x, y).$$

The feature embedding $x \mapsto k(x, \cdot)$ induced by a kernel therefore preserves the distances d_k. A useful aspect of kernel methods, is that they allow a post-analysis based on the embedded features, see also Section 2 in S5 Appendix.

A partial reverse implication is also true. For a particular type of semi-metric d on $\mathcal{X}$ (these metrics are called Hilbertian, see S5 Appendix) it is possible to construct a kernel k on $\mathcal{X}$ defined for all $x, y \in \mathcal{X}$ by

$$k(x,y) = -\tfrac{1}{2}d^2(x,y) + \tfrac{1}{2}d^2(x,x_0) + \tfrac{1}{2}d^2(x_0,y),$$

where $x_0 \in \mathcal{X}$ is an arbitrary reference point, such that the distance in the corresponding RKHS $\mathcal{H}_k$ is d.

Kernels can be shifted in such a way that the origin in the induced RKHS changes but the metric in (A) remains fixed (see Lemma 1.1 in S5 Appendix). A natural origin in the simplex is given by the point $u = (\frac{1}{p}, \ldots, \frac{1}{p})$, therefore we have shifted all kernels such that $k(u, \cdot) \equiv 0$ and hence correspond to the origin in $\mathcal{H}_k$. In S5 Appendix, we provide a short overview of the mathematical results that connect kernels and metrics.

B Weighted kernels - including prior information

In this section, we discuss how to include prior knowledge, e.g. phylogenetic information, into the simplex kernels. We assume the information is encoded in a matrix $W \in \mathbb{R}^{p \times p}$ where each element corresponds to a measure of similarity between components. That is, $W_{i,j}$ is large if components i and j are similar (or related) and small otherwise. We assume that W is symmetric, positive semi-definite and all entries in W are non-negative.

The linear kernel and all kernels based on probability distributions have the form

$$k(x,y) = \textstyle\sum_{i=1}^{p} k_0(x^i, y^i) \tag{B}$$

and we therefore define the weighted version by

$$k_W(x,y) = \sum_{j,\ell=1}^{p} W_{j,\ell} \cdot k_0(x^j, y^\ell). \tag{C}$$

The weighted versions of the remaining kernels are defined individually. A full list of the weighted kernels is given in Section 2 of S7 Appendix.

B.1 Validity of weighted kernels

In order to use the proposed weighted kernels, we need to ensure that they are indeed positive definite. In the following, we prove this for the weighted versions of the *linear kernel*, the *Hilbertian kernel*, the *Generalized-JS kernel*, the *RBF kernel* and the *Aitchison kernel*. We do not prove it for the *Aitchison RBF kernel* and the *Heat Diffusion kernel* and only note that they appear to be positive definite from our empirical evaluations.

We begin by showing that the kernel defined in (C) is positive definite whenever $k_0 : [0,1] \times [0,1] \to \mathbb{R}$ is positive definite. To see this, fix $x_1, \dots, x_n \in \mathbb{S}^{p-1}$ and $\alpha \in \mathbb{R}^n$ and denote by $K_W \in \mathbb{R}^{n \times n}$ the kernel Gram-matrix based on $x_1, \dots, x_n$ and kernel k_W. Then,

$$\begin{aligned} \alpha^\top K_W \alpha &= \sum_{i,r=1}^{n} \sum_{j,\ell=1}^{p} \alpha_i \alpha_r W_{j,\ell} k_0(x_i^j, x_r^\ell) \\ &= \sum_{j,\ell=1}^{p} W_{j,\ell} \left(\sum_{i,r=1}^{n} \alpha_i \alpha_r k_0(x_i^j, x_r^\ell) \right). \end{aligned}$$

Since k_0 is positive definite, it holds that $\sum_{i,r} \alpha_i \alpha_r k_0(x_i^j, x_r^\ell) \geq 0$ and hence $\alpha^\top K_W \alpha \geq 0$ since all entries in W are non-negative.

We now go over the individual weighted kernels and argue that they are positive definite.

- **Linear kernel** Since $\mathbb{R}$ is a Hilbert space with the inner product xy which induces the $|x-y|$ it follows that the squared distance $d^2_{\text{Linear}}(x,y) := (x-y)^2$ is Hilbertian as well. Applying Theorem 1.1 in S5 Appendix we know that the distance is of negative type. Thus, based on the one-dimensional squared linear distance d^2_{Linear}, we apply Theorem 1.2 in S5 Appendix with $x_0 = \frac{1}{p}$ to construct the following positive definite kernel k_0 defined for all $x, y \in [0,1]$ by

  $$k_0(x,y) := -\tfrac{1}{2}(x-y)^2 + \tfrac{1}{2}(x - \tfrac{1}{p})^2 + \tfrac{1}{2}(\tfrac{1}{p} - y)^2 = xy - \tfrac{x}{p} - \tfrac{y}{p} + \tfrac{1}{p^2}.$$

  Comparing this with our weighted linear kernel in Section 2 of S7 Appendix, we see that the weighted linear kernel has the form (C) and is therefore positive definite by the above argument.

- **Hilbertian kernel** As shown by [1] the distance $d_{\text{Hilbert}} : \mathbb{R}_+ \times \mathbb{R}_+ \to \mathbb{R}$ defined for all $x, y \in \mathbb{R}_+$ by

$$d^2_{\text{Hilbert}}(x,y) = \frac{2^{\frac{1}{b}}\left[x^a + y^a\right]^{\frac{1}{a}} - 2^{\frac{1}{a}}\left[x^b + y^b\right]^{\frac{1}{b}}}{2^{\frac{1}{a}} - 2^{\frac{1}{b}}}$$

is a Hilbertian metric on $\mathbb{R}_+$. Applying Theorem 1.2 in S5 Appendix with $x_0 = \frac{1}{p}$ results in a positive definite kernel k_0 that when combined as in (C) results in the proposed weighted Hilbertian kernels in Section 2 of S7 Appendix. Therefore, we have shown that the weighted Hilbertian kernels are positive definite as long as W has non-negative entries.

- **Generalized-JS kernel** Similarly the weighted Generalized-JS kernels in Section 2 of S7 Appendix can all be decomposed as in (C) with a one-dimensional kernels k_0 on $[0, 1]$. [2] show that all these k_0 can be generated using Theorem 1.2 in S5 Appendix with $x_0 = \frac{1}{p}$ based on Hilbertian metrics. Hence, all weighted Generalized-JS kernels are positive definite as long as W has non-negative entries.

- **Aitchison kernel** To show that the weighted Aitchison kernel (defined in Section 2 of S7 Appendix) is positive definite, we first define the mapping $\Phi : \mathbb{S}^{p-1} \to \mathbb{R}^p$ by $\Phi(x) := \frac{x+c}{g(x+c)}$. Then, the weighted Aitchison kernel is given by

$$k(x, y) = \Phi(x)^\top W \Phi(y).$$

Since W is symmetric and positive semi-definite there exists $M \in \mathbb{R}^{p \times p}$ such that $W = M^\top M$. Therefore, for any $\alpha \in \mathbb{R}^n$ and $x_1, \dots, x_n \in \mathbb{S}^{p-1}$ it holds that

$$\sum_{i,r} \alpha_i \alpha_r k(x_i, x_r) = \sum_{i,r} \alpha_i \alpha_r (M\Phi(x_i))^\top M\Phi(x_r) \geq 0.$$

Hence, k is positive definite.

- **RBF kernel** Using the symmetry of W the weighted RBF kernel can be expressed as

follows

$$
\begin{aligned}
k(x,y) &= \exp\Big(-\frac{1}{\sigma^2}\sum_{j,\ell=1}^{p} W_{j,\ell}(x^j - y^\ell)^2\Big)\\
&= \underbrace{\exp\Big(-\frac{1}{\sigma^2}\sum_{j,\ell=1}^{p} W_{j,\ell}(x^j)^2\Big)\exp\Big(-\frac{1}{\sigma^2}\sum_{j,\ell=1}^{p} W_{j,\ell}(y^j)^2\Big)}_{=:A(x,y)}\\
&\quad \underbrace{\cdot\exp\Big(\frac{2}{\sigma^2}\sum_{j,\ell=1}^{p} W_{j,\ell}x^j y^\ell\Big)}_{=:B(x,y)}
\end{aligned}
$$

The function A is a positive definite kernel since it is the inner-product of a feature mapping. The function B can be shown to be a kernel by considering the Taylor expansion of the exponential function and using that sums and limits of positive definite kernels are again positive definite together with the fact that W is positive semi-definite. Therefore, the weighted RBF kernel is positive definite.

C UniFrac-Weighting

In this section, we show how prior information based on the UniFrac-Distance [3] can be encoded into a weight matrix $W \in \mathbb{R}^{p\times p}$. Depending on the application at hand different distances can be used in a similar way. The UniFrac-Distance is a β-diversity measure that uses phylogenetic information to compare two compositional samples $x, y \in \mathbb{S}^{p-1}$. Each element of the sample is hereby placed on a phylogenetic tree. The distance between both samples is computed via quantification of overlapping branch length, that is,

$$
\text{UniFrac-Distance}(x,y) = \frac{\text{sum of unshared branch length of } x \text{ and } y}{\text{sum of all tree branch length of } x \text{ and } y} \in [0,1].
$$

Based on the UniFrac-Distance, we define two similarity matrices $M^A, M^B \in [0,1]^{p\times p}$ for all $i,j \in \{1,\dots,p\}$ by

$$M^A_{i,j} \coloneqq 1 - \text{UniFrac-Distance}(e_i, e_j),$$
$$M^B_{i,j} \coloneqq \sum_{\ell=1}^{p} \text{UniFrac-Distance}(e_i, e_\ell) \cdot \text{UniFrac-Distance}(e_j, e_\ell),$$

where $e_i, e_j \in \mathbb{S}^{p-1}$ with 1 on the i-th and j-th coordinate, respectively. M^A and M^B are two options of encoding the UniFrac-Distance as a similarity. M^B is positive semi-definite by construction, while this is not true for M^A and should be checked empirically. We recommend using M^A whenever it is positive semi-definite.

We then construct the weight matrix $W^{\text{UniFrac}} \in \mathbb{R}^{p\times p}$ by scaling M^* such that the diagonal entries are one, that is,

$$W^{\text{UniFrac}} \coloneqq DM^*D,$$

where $D = \text{diag}(\sigma_1, \dots, \sigma_p)$, with $\sigma_i = 1/\sqrt{M^*_{i,i}}$. Since by construction the matrix M^* has its largest values on the diagonal, this weight matrix takes values in $[0,1]$. Moreoever, by construction it remains symmetric and positive semi-definite.

References

1. Hein M, Bousquet O. Hilbertian metrics and positive definite kernels on probability measures. In: International Workshop on Artificial Intelligence and Statistics. PMLR; 2005. p. 136–143.

2. Topsøe F. Jenson-Shannon divergence and norm-based measures of discrimination and variation; 2003. Available from: `https://web.math.ku.dk/~topsoe/sh.ps`.

3. Lozupone C, Knight R. UniFrac: a new phylogenetic method for comparing microbial communities. Applied and Environmental Microbiology. 2005;71(12):8228–8235.
