# Supplementary material for: Supervised learning and model analysis with compositional data
Source: PLoS Comput Biol. 2023 Jun 30;19(6):e1011240. doi: 10.1371/journal.pcbi.1011240 (PMC10343141; doi:10.1371/journal.pcbi.1011240)
Supplement: S3 Appendix — Datasets pre-processing, parameter setup, construction of the weighting matrices with UniFrac-distance and further experiment results based on the cirrhosis and centralparksoil datasets. (PDF) [file pcbi.1011240.s003.pdf]

S3 Appendix: Details and additional results for experiments

A List of datasets

Dataset	Reference	Prediction tasks	Dim ($n \times d$)	Additional preprocessing
rmp	[1]	classification:<br>- Crohn's disease vs healthy	95×351	none
camp	[2]	classification:<br>- parasite infected vs healthy	270×622	none
cirrhosis	[3]	classification:<br>- cirrhosis vs healthy	130×444	aggregated to species & prev./abun. filtering
cancer	[4]	classification:<br>- cancer vs non-cancer	490×335	none
impaired-diabetes	[5]	classification:<br>- impaired vs type 2 diabetes	101×3758	none
nugent-category	[6]	classification:<br>- nugent score high vs low	342×305	none
gastro-oral	[7]	classification:<br>- gastrointestinal vs oral	2070×1218	none
healthy-cd	[8]	classification:<br>- healthy vs Crohn's disease	74×367	none
kostic	[9]	classification:<br>- healthy vs tumor	172×409	none
malawi-venezuela	[10]	classification:<br>- Malawi vs Venezuela	54×1544	none
black-hispanic	[6]	classification:<br>- black vs Hispanic	199×305	none
ss-paired	[7]	classification:<br>- sub vs supragingival plaque	408×1218	none
usa-malawi	[10]	classification:<br>- US vs Malawi	150×1544	none
st-paired	[7]	classification:<br>- stool vs tongue dorsum	404×1218	none
gevers_ileum	[11]	classification:<br>- Crohn's disease vs healthy	140×446	none
yatsunenko_sex	[10]	classification:<br>male vs female	129×1544	none
normal-diabetes	[5]	classification:<br>- normal vs type 2 diabetes	96×3758	none
healthy-uc	[8]	classification:<br>- healthy vs Ulcerative colitis	59×367	none

hmp_sex	[7]	classification:<br>- female vs male	180×1218	none
qin2012	[12]	classification:<br>- healthy vs type 2 diabetes	124×2526	none
turnbaugh	[13]	classification:<br>- lean vs obese	142×232	none
gevers_rectum	[11]	classification:<br>- Crohn's disease vs health	160×446	none
qin2014	[3]	classification:<br>- cirrhosis vs healthy	130×2579	none
white-black	[6]	classification:<br>- white vs black	200×305	none
centralparksoil	[14]	regression:<br>- ph level of soil	580×1498	prev./abun. filtering
uk	[15]	regression:<br>- BMI	882×327	UK subpopulation & prev./abun. filtering
hiv	[16]	regression:<br>- CD4+ cell counts	152×282	none
tara	[17]	regression:<br>- ocean salinity	136×2407	prev./abun. filtering
ravel_ph	[6]	regression:<br>- vaginal pH	388×305	none
pcdai-rectum	[11]	regression:<br>- PCDAI scores	51×446	none
pcdai-ileum	[11]	regression:<br>- PCDAI scores	67×446	none
baby-age	[10]	regression:<br>- infant age	49×1544	none
nugent-score	[6]	regression:<br>- nugent score	388×305	none

Table A. List of microbiome datasets used to benchmark `KernelBiome`. The cirrhosis dataset is a processed version of qin2014. Whenever prevalence/abundance filtering (prev./abun. filtering) is applied it means that only taxa that appear in 25% of the samples and with a median non-zero count of 5. Datasets other than the following ones are taken from the MLRepo [18] are taken directly without further processing: uk, camp, centralparksoil, cirrosis, cancer, hiv, rmp, tara.

B Weighting matrix for weighted `KernelBiome`

The weight matrix W^{UniFrac} for the cirrhosis dataset [3] and the centralparksoil dataset [14] are presented as heatmaps in Fig A. Using our proposed weighted kernels (see Section 2 of S7 Appendix) with the UniFrac-based weight matrix W^{UniFrac} is different from incorporating the UniFrac-distance via kernel convolution as proposed by [19].

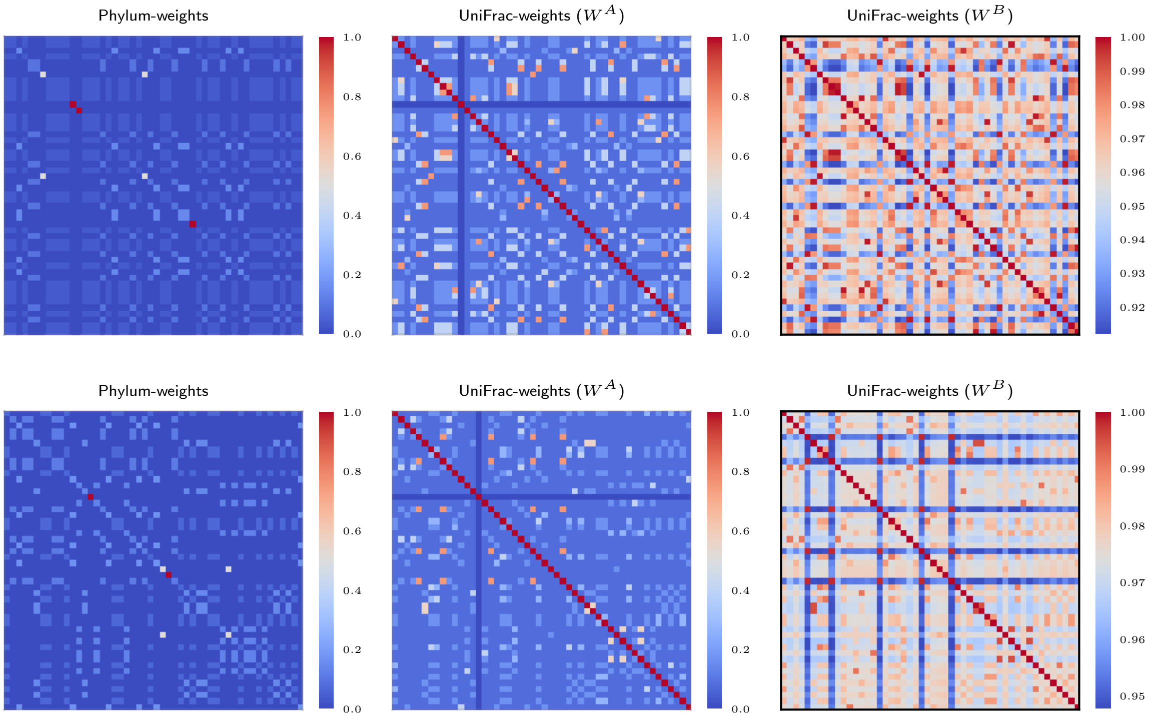

Fig A. Visualization of the phylum-weights and the two UniFrac-weights $W^A = DM^A D$ for $W^B = DM^B D$, based on the 50 pre-screened species (see S3 Appendix). Upper panel: cirrhosis dataset. Lower panel: centralparksoil dataset.

C Detailed experiment results on public datasets

C.1 Prediction performance

Here we provide more details on the the prediction performance evaluation. Boxplots of prediction scores for all 33 datasets as in Fig 3 in the main text are given in Fig B, and PR curves accompanying the boxplots can be found in Fig C. We can see that `KernelBiome` achieves the best results for most of the tasks. For classification tasks, `KernelBiome` performs competitively both in terms of balanced accuracy and PR curves. (For `SVM-RBF`, `KB-Aitchison` and `KernelBiome`, the PR curves are based on the estimated probabilities computed in the sklearn-package. We observed a slight mismatch between these predicted probabilities and predicted classes in some of the examples, which is due to a bug `https://github.com/scikit-learn/scikit-learn/issues/13211`. We therefore recommend putting more emphasis on the accuracy plots.) The frequency of kernels selected the most often by `KernelBiome` is given in Table. B.

Dataset (short name)	Kernel	Frequency (%)
rmp	aitchison	59.5
camp	aitchison-rbf	44.5
cirrhosis	aitchison-rbf	65.5
cancer	aitchison	73.5
impaired-diabetes	aitchison	54.0
nugent-category	aitchison-rbf	63.0
gastro-oral	aitchison	100.0
healthy-cd	aitchison-rbf	46.0
kostic	aitchison-rbf	55.5
malawi-venezuela	aitchison	100.0
black-hispanic	aitchison	55.5
ss-paired	aitchison-rbf	78.5
usa-malawi	aitchison	100.0
st-paired	aitchison	100.0
gevers_ileum	generalized-js	29.5
yatsunenko_sex	aitchison-rbf	70.5
normal-diabetes	aitchison-rbf	48.0
healthy-uc	aitchison-rbf	48.5
hmp_sex	aitchison-rbf	35.5
qin2012	aitchison	53.0
turnbaugh	aitchison	56.5
gevers_rectum	aitchison-rbf	53.5
qin2014	aitchison-rbf	81.5
white-black	aitchison	50.0
centralparksoil	generalized-js	45.0
uk	aitchison-rbf	100.0
hiv	aitchison-rbf	97.0
tara	aitchison-rbf	93.0
ravel_ph	heat-diffusion	61.0
pcdai-rectum	rbf	67.0
pcdai-ileum	rbf	40.0
baby-age	aitchison	91.0
nugent-score	aitchison-rbf	99.5

Table B. Kernels selected most frequently by `KernelBiome` for all 33 datasets.

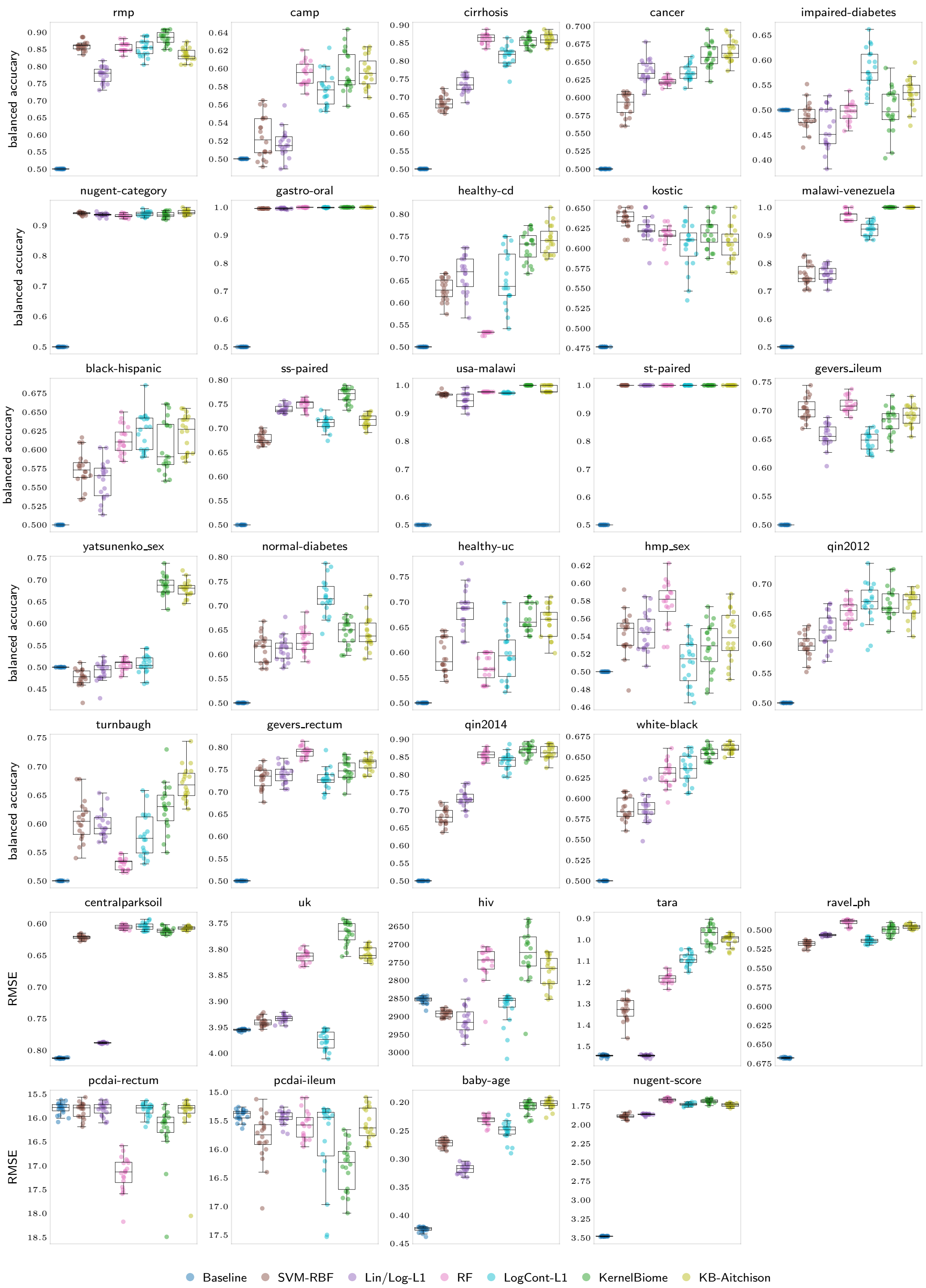

Fig B. Comparison of predictive performance on the 33 public datasets based on a 10-fold train/test split.

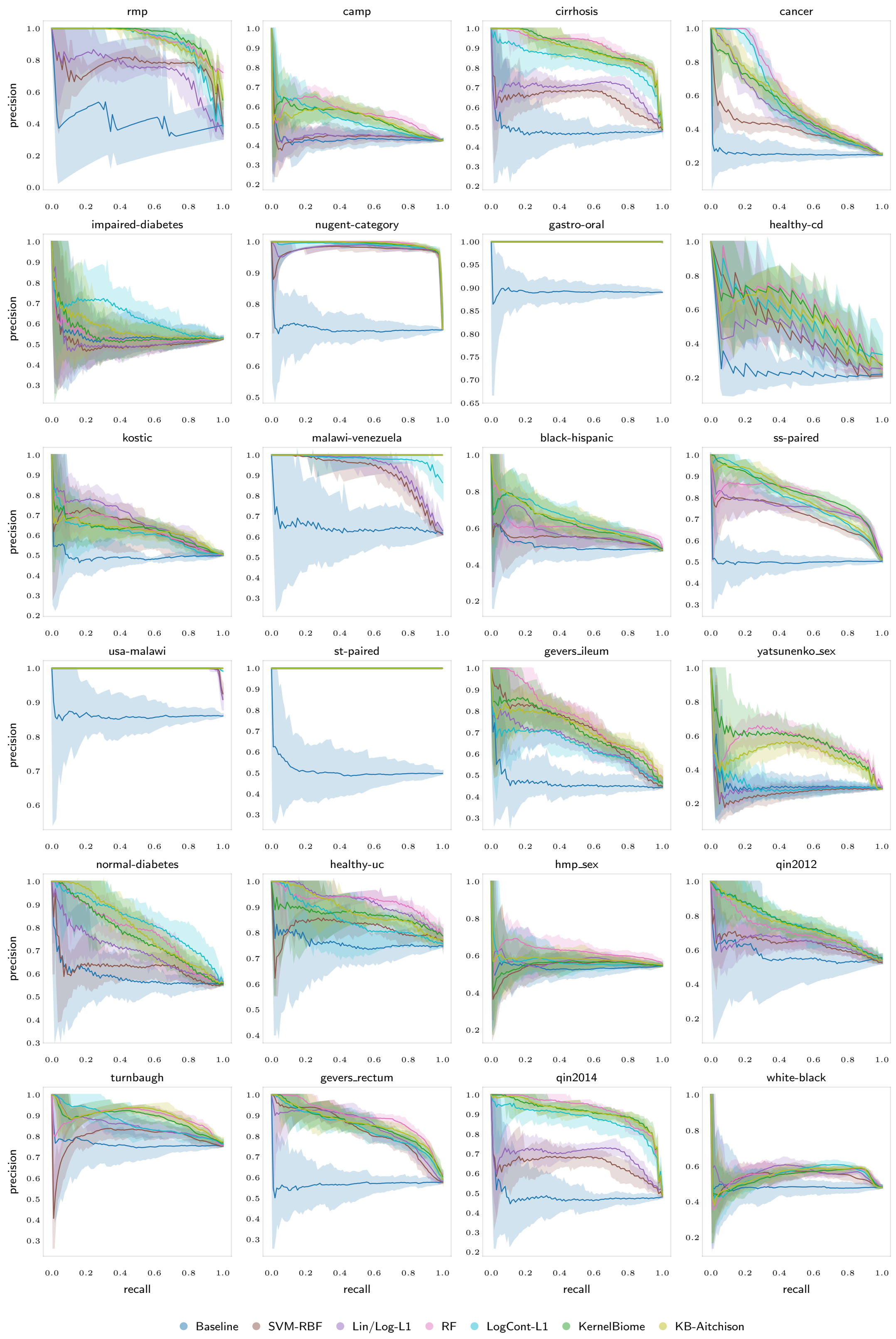

Fig C. PR curves for the 24 classification datasets. The solid curve is the average curve from the 20 random 10-fold CV, and the shaded area is the 95% confidence band.

C.2 Model analysis

Here we include the remaining model analysis results for the cirrhosis and centralparksoil datasets. As in the main paper, we screened both data sets to only include the 50 taxa with the highest absolute CFIs by `KernelBiome` with Aitchison kernel. Kernel PCA plots for the cirrhosis dataset is given in Fig D and the CFI values for the centralparksoil dataset are given in Fig E.

Furthermore, we also provide the missing circle plots here. The extended version of Fig 5C in the main text with long labels is given in Fig F. Fig G is the circle plot for the cirrhosis dataset based on the generlized-JS kernel. Fig H is the circle plot for the centralparksoil dataset based on the Aitchison kernel.

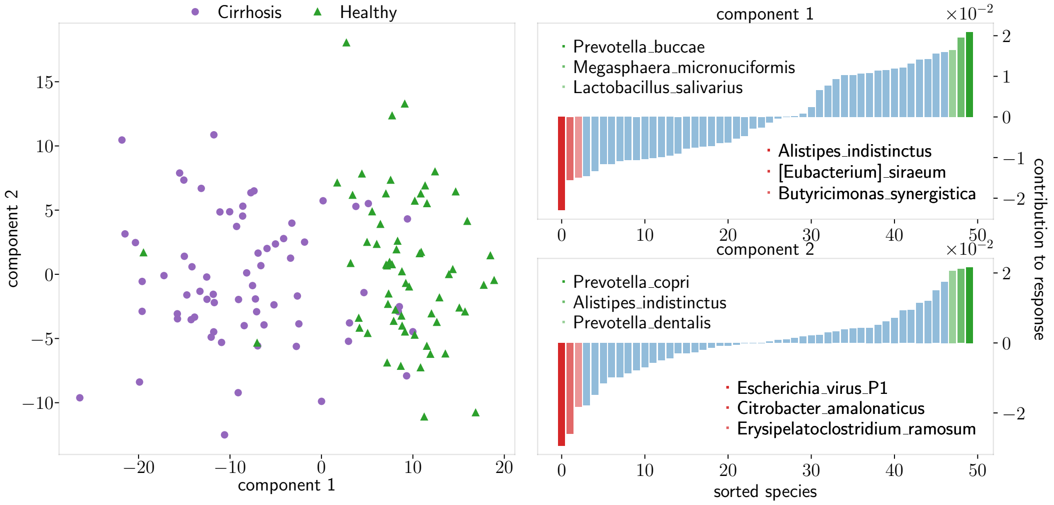

Fig D. Kernel PCA plot and contributions of the 50 taxa to component 1 and 2 sorted from the most negative contribution to the most positive contribution (cirrohsis).

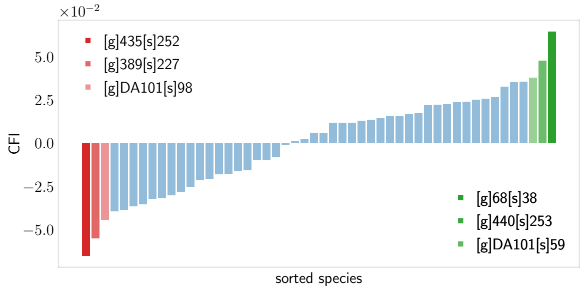

Fig E. CFI values for the 50 taxa sorted from the most negative contribution to the most positive contribution to the response (centralparksoil).

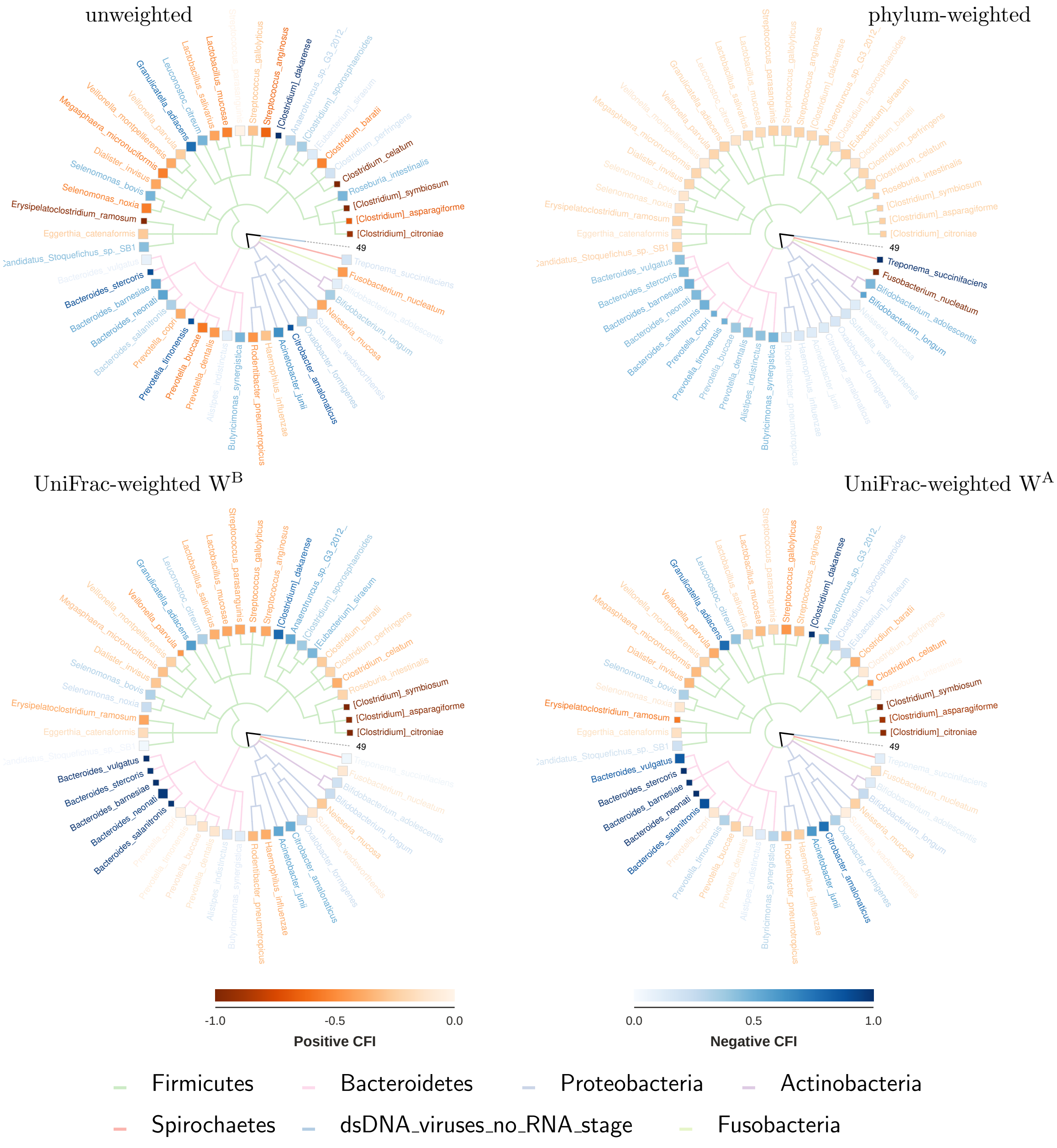

Fig F. Scaled CFI values for cirrhosis dataset where a darker color shade of the name of the microbiota signifies a stronger (positive resp. negative) feature influence (Aitchison Kernel).

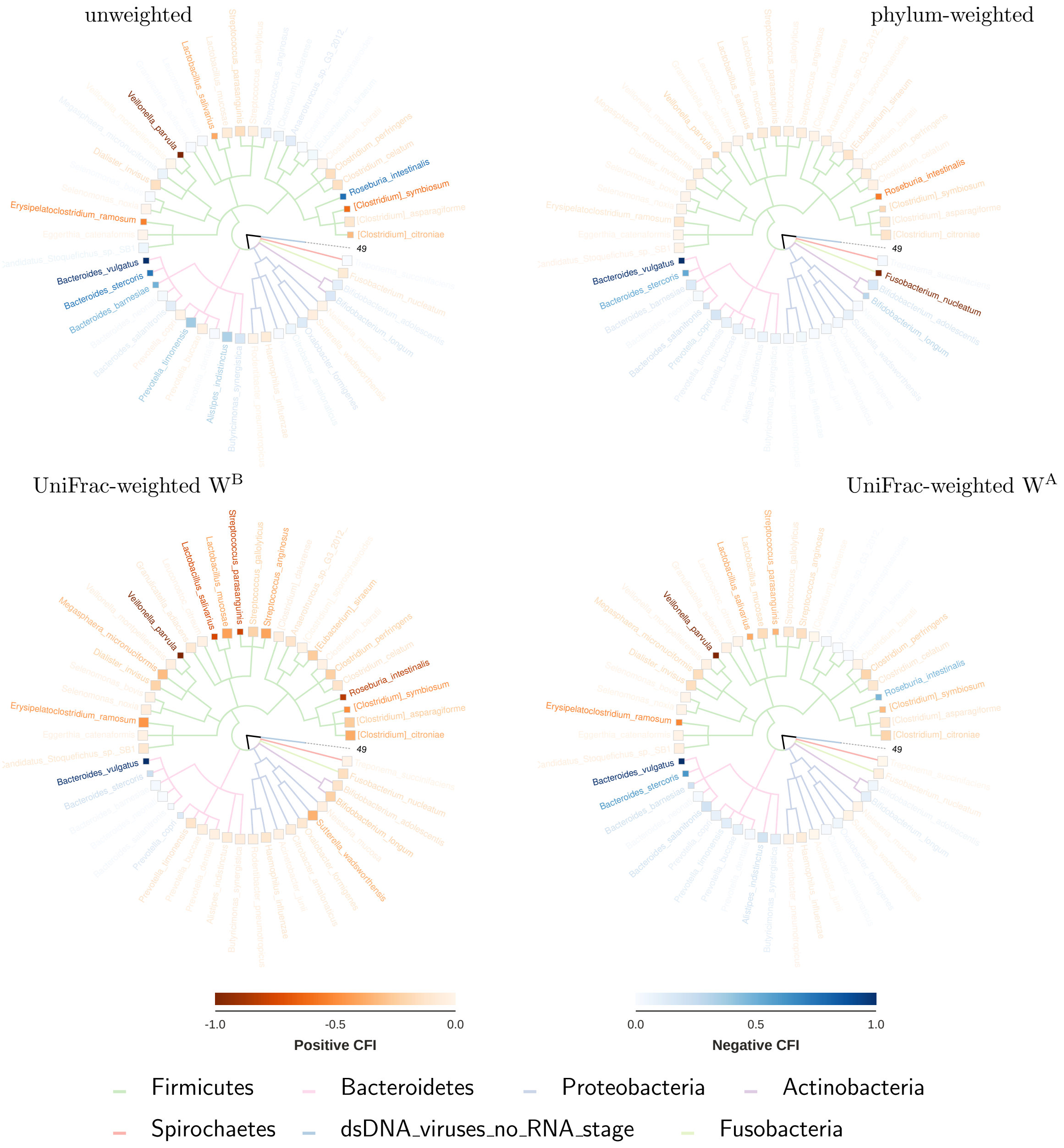

Fig G. Scaled CFI values for cirrhosis dataset where a darker color shade of the name of the microbiota signifies a stronger (positive resp. negative) feature influence (Generalized JS Kernel).

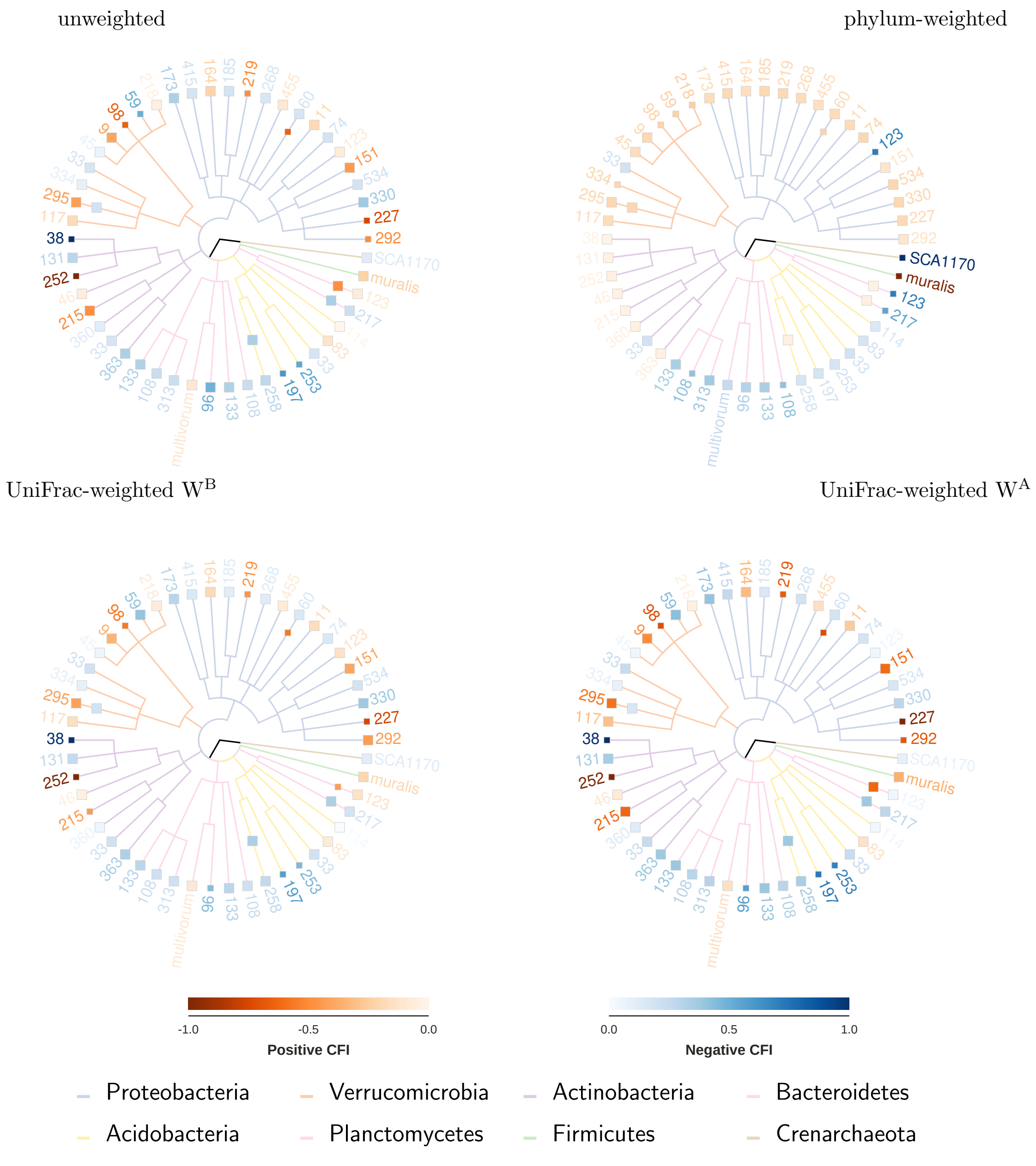

Fig H. Scaled CFI values for centralparksoil dataset where a darker color shade of the name of the microbiota signifies a stronger (positive resp. negative) feature influence (Aitchison Kernel).

References

1. Vandeputte D, Kathagen G, D'hoe K, Vieira-Silva S, Valles-Colomer M, Sabino J, et al. Quantitative microbiome profiling links gut community variation to microbial load. Nature. 2017;551(7681):507–511.

2. Berry AS, Johnson K, Martins R, Sullivan MC, Farias Amorim C, Putre A, et al. Natural infection with Giardia is associated with altered community structure of the human and canine gut microbiome. Msphere. 2020;5(4):e00670–20.

3. Qin N, Yang F, Li A, Prifti E, Chen Y, Shao L, et al. Alterations of the human gut microbiome in liver cirrhosis. Nature. 2014;513(7516):59–64.

4. Baxter NT, Ruffin MT, Rogers MA, Schloss PD. Microbiota-based model improves the sensitivity of fecal immunochemical test for detecting colonic lesions. Genome medicine. 2016;8(1):1–10.

5. Karlsson FH, Tremaroli V, Nookaew I, Bergström G, Behre CJ, Fagerberg B, et al. Gut metagenome in European women with normal, impaired and diabetic glucose control. Nature. 2013;498(7452):99–103.

6. Ravel J, Gajer P, Abdo Z, Schneider GM, Koenig SS, McCulle SL, et al. Vaginal microbiome of reproductive-age women. Proceedings of the National Academy of Sciences. 2011;108(supplement_1):4680–4687.

7. Human Microbiome Project Consortium. Structure, function and diversity of the healthy human microbiome. nature. 2012;486(7402):207–214.

8. Morgan XC, Tickle TL, Sokol H, Gevers D, Devaney KL, Ward DV, et al. Dysfunction of the intestinal microbiome in inflammatory bowel disease and treatment. Genome biology. 2012;13(9):1–18.

9. Kostic AD, Gevers D, Pedamallu CS, Michaud M, Duke F, Earl AM, et al. Genomic analysis identifies association of Fusobacterium with colorectal carcinoma. Genome research. 2012;22(2):292–298.

10. Yatsunenko T, Rey FE, Manary MJ, Trehan I, Dominguez-Bello MG, Contreras M, et al. Human gut microbiome viewed across age and geography. nature. 2012;486(7402):222–227.

11. Gevers D, Kugathasan S, Denson LA, Vázquez-Baeza Y, Van Treuren W, Ren B, et al. The treatment-naive microbiome in new-onset Crohn's disease. Cell host & microbe. 2014;15(3):382–392.

12. Qin J, Li Y, Cai Z, Li S, Zhu J, Zhang F, et al. A metagenome-wide association study of gut microbiota in type 2 diabetes. Nature. 2012;490(7418):55–60.

13. Turnbaugh PJ, Ley RE, Hamady M, Fraser-Liggett CM, Knight R, Gordon JI. The human microbiome project. Nature. 2007;449(7164):804–810.

14. Ramirez KS, Leff JW, Barberán A, Bates ST, Betley J, Crowther TW, et al. Biogeographic patterns in below-ground diversity in New York City's Central Park are similar to those observed globally. Proceedings of the Royal Society B: Biological Sciences. 2014;281(1795):20141988.

15. McDonald D, Hyde E, Debelius JW, Morton JT, Gonzalez A, Ackermann G, et al. American gut: an open platform for citizen science microbiome research. Msystems. 2018;3(3):e00031–18.

16. Rivera-Pinto J, Egozcue JJ, Pawlowsky-Glahn V, Paredes R, Noguera-Julian M, Calle ML. Balances: a new perspective for microbiome analysis. MSystems. 2018;3(4):e00053–18.

17. Sunagawa S, Acinas SG, Bork P, Bowler C, Eveillard D, Gorsky G, et al. Tara Oceans: towards global ocean ecosystems biology. Nature Reviews Microbiology. 2020;18(8):428–445.

18. Vangay P, Hillmann BM, Knights D. Microbiome Learning Repo (ML Repo): A public repository of microbiome regression and classification tasks. Gigascience. 2019;8(5):giz042.

19. Zhao N, Chen J, Carroll IM, Ringel-Kulka T, Epstein MP, Zhou H, et al. Testing in microbiome-profiling studies with MiRKAT, the microbiome regression-based kernel association test. The American Journal of Human Genetics. 2015;96(5):797–807.
