# Supplementary material for: Supervised learning and model analysis with compositional data
Source: PLoS Comput Biol. 2023 Jun 30;19(6):e1011240. doi: 10.1371/journal.pcbi.1011240 (PMC10343141; doi:10.1371/journal.pcbi.1011240)
Supplement: S4 Appendix — Consistency of CFI and CPD and comparison of CFI and CPD with their non-simplex counterparts. (PDF) [file pcbi.1011240.s004.pdf]

S4 Appendix: Additional experiments with simulated data

A Consistency of CPD and CFI

We illustrate the consistency of CPD and CFI from Theorem 2.1 in the main text based on `KernelBiome` with the following example. Let k_{tv} be the total variation kernel and consider the function

$$f : x \mapsto 100 \cdot k_{\mathrm{tv}}(z, x)$$

with

$$z = (0.06544714, 0.08760064, 0.17203408, 0.07502236, 0.1642615,$$
$$0.03761901, 0.18255478, 0.13099514, 0.08446536) \in \mathbb{S}^8$$

being a fixed and randomly selected point. Furthermore, we generate an i.i.d. dataset (X_1, Y_1), $\ldots, (X_n, Y_n)$ based on the following 2 step generative model.

- **Step 1:** Generate a random variable $\tilde{X} = (\tilde{X}^1, \ldots, \tilde{X}^9)$ such that the three blocks $(\tilde{X}^1, \tilde{X}^2, \tilde{X}^3)$, $(\tilde{X}^4, \tilde{X}^5, \tilde{X}^6)$, and $(\tilde{X}^7, \tilde{X}^8, \tilde{X}^9)$ are i.i.d. from LogNormal$(0, \Sigma)$, where $\Sigma = \begin{pmatrix} 1 & 0.25 & -0.25 \\ 0.25 & 1 & 0.25 \\ -0.25 & 0.25 & 1 \end{pmatrix}$. Then, X_i is constructed by normalizing $\tilde{X}$, that is, $X_i = \tilde{X} / \sum_{j=1}^{9} \tilde{X}^j$. The block structure adds non-trivial correlation structure between the compositional components.

- **Step 2:** Generate Y_i based on X_i by

$$Y_i = f(X_i) + \epsilon_i$$

  with $\epsilon_i \overset{iid}{\sim} \mathcal{N}(0, 1)$.

Based on one such dataset, we estimate the CFI and CPD for a fitted `KernelBiome` estimator (using kernel ridge regression and default settings), and compare the estimates against the

population CFI and CPD calculated from the true function f. In Fig A, we report the mean squared deviations (MSD) for both CFI and CPD based on 100 such datasets for each sample size.

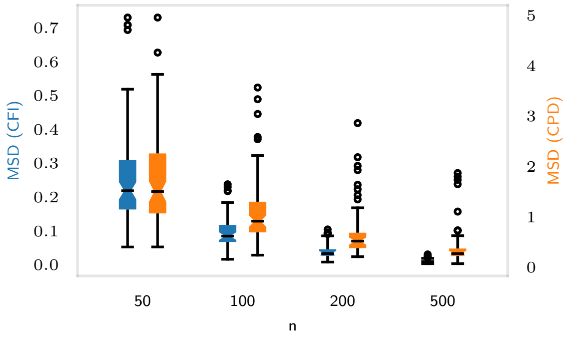

Fig A. MSD of estimated CFI and CPD using `KernelBiome` estimator based on 100 random datasets for each sample size. For CPD, we calculate the true and estimated CPD based on 100 evenly spaced grid points within the range of $[0.001, 0.999]$ and the reported MSD is the average MSD over the 9 components. As the sample size n increases the CFI and CPD estimates based on `KernelBiome` converge to the true population quantities.

B Comparing CFI and CPD with permutation importance and partial dependence plots

Two common approaches to assess the importance of individual features are permutation importance (PI) and partial dependency plot (PDP). PI of the j-th feature is defined as the mean difference between the baseline mean squared error of a fitted model and the average mean squared error after permuting the j-th feature column a certain number of times. PDP is used to describe how individual features contribute to a fitted model. For the j-th feature, it describes its contribution by the function $z \mapsto \mathbb{E}[\hat{f}(X^1, \ldots, X^{j-1}, z, X^j, \ldots, X^p)]$, where $\hat{f}$ is the fitted model. Both PI and PDP can be misleading when used with compositional covariates.

In this section, we illustrate this based on two examples. In both cases, the proposed adjusted measures CFI and CPD remain correct, while the PI and PDP are incorrect. Consider

	f_1			f_2		
	x^1	x^2	x^3	x^1	x^2	x^3
CFI	0.85	0.87	**-1.72**	1.94	-1.94	**0.00**
RI	3.76	2.99	**0.00**	0.00	-4.72	**-4.40**
PI	11.66	5.76	**0.00**	0.00	28.98	**24.72**

Table A. CFI, RI and PI for the two functions f_1 and f_2 defined in (A). Only CFI correctly attributes the effect of x^3 (marked in bold).

the two functions

$$\begin{aligned} f_1 &: x \mapsto 10x^1 + 10x^2 \\ f_2 &: x \mapsto \frac{1 - x^2 - x^3}{1 - x^3}. \end{aligned} \tag{A}$$

For f_1, changes in all coordinates affect the function value due to the simplex constraint. For f_2, only changes in x^1 and x^2 affect the function value but not changes in x^3. This is because on the simplex $f_2(x) = \frac{x^1}{x^1+x^2}$. An importance measure should therefore associate a non-zero value to x^3 for f^1 and zero to x^3 for f^2.

We generate 200 i.i.d. observations $X_1 \ldots, X_{100}$ with $X_i \overset{d}{=} \tilde{X}_i / \sum_{j=1}^{3} \tilde{X}_i^j$ for $\tilde{X}_i \overset{\text{i.i.d.}}{\sim}$ LogNormal$(0, \text{Id}_3)$ (LogNormal(μ, Σ) denotes the log-normal distribution with location parameter μ and scale parameter Σ. Id_3 denotes the 3-dimensional identity matrix.) and compute PI, PDP, CFI and CPD for each of the two functions. The results are given in Table A and Fig B.

As expected, the CFI and also CPD correctly capture the behavior of the two functions. However, PI and PDP are incorrect in both cases: For f_1 the variable x^3 shows no effect both with PI and PDP and for f_2 the variable x^3 is falsely assigned a strong negative effect even though it does not affect the function value at all. In Table A, we have additionally computed the relative influence (RI) given by $\mathbb{E}[\frac{d}{dx^j}\hat{f}(X)]$ due to [1]. It has the same problems as PI as it does not take into account the simplex structure.

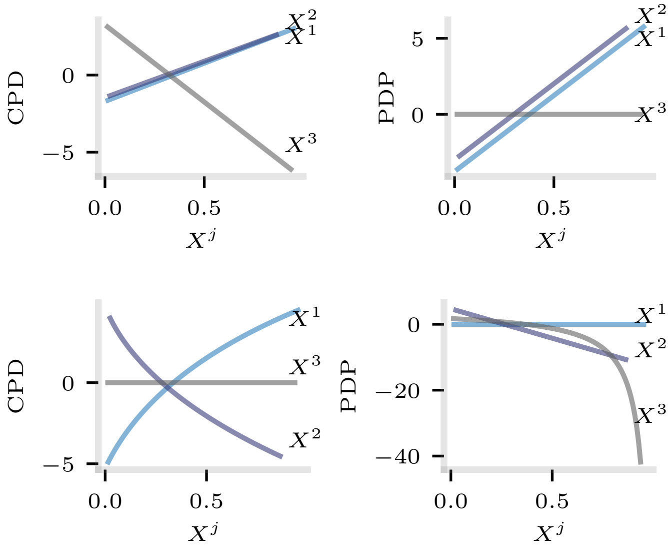

Fig B. Top row: CPD and PDP plot based on f_1. Bottom row: CPD and PDP plot based on f_2. CPD reflects the true feature importance on the simplex while PDP does not.

References

1. Friedman JH. Greedy function approximation: a gradient boosting machine. Annals of Statistics. 2001; p. 1189–1232.
