# Supplementary material for: Supervised learning and model analysis with compositional data
Source: PLoS Comput Biol. 2023 Jun 30;19(6):e1011240. doi: 10.1371/journal.pcbi.1011240 (PMC10343141; doi:10.1371/journal.pcbi.1011240)
Supplement: S5 Appendix — Mathematical background on kernels and details on dimensionality and visualization with kernels. (PDF) [file pcbi.1011240.s005.pdf]

S5 Appendix: Background on kernels

A Connection between metrics and kernels

Definition A.1 (Metric, semi-metric, and quasi-metric)**.** A function $d : \mathcal{X} \times \mathcal{X} \to \mathbb{R}$ is called a *metric* if it satisfies

(a) $d(x, x) = 0$,

(b) $d(x, y) = d(y, x) \geq 0$,

(c) $d(x, y) \leq d(x, z) + d(y, z)$,

(d) $d(x, y) = 0 \Rightarrow x = y$.

It is called a *semi-metric* if it satisfies (a)-(c), and a *quasi-metric* if it satisfies (a)-(b).

Definition A.2 (Function of negative type and Hilbertian metric)**.** A quasi-metric $d : \mathcal{X} \times \mathcal{X} \to \mathbb{R}$ is called of *negative-type* if for all $n \in \mathbb{N}$, all $x_1, \cdots, x_n \in \mathcal{X}$, and all $c_1, \cdots, c_n \in \mathbb{R}$ with $\sum_{i=1}^n c_i = 0$, it holds that

$$\sum_{i,j=1}^{n} c_i c_j d^2(x_i, x_j) \leq 0. \tag{A}$$

If d is a (semi-)metric, then d is also called *Hilbertian.*

Theorem A.1 (Sufficient and necessary conditions for isometric embeddings)**.** A quasi-metric space (X, d) can be isometrically embedded in a Hilbert space if and only if d is of negative type.

Proof. See [1, Theorem 2.4]. □

Definition A.3 ((conditionally) positive definite kernels)**.** A symmetric function $k : \mathcal{X} \times \mathcal{X} \to \mathbb{R}$ (i.e., $\forall x, y \in \mathcal{X}, k(x, y) = k(y, x)$) is called a *positive definite kernel* if and only if for all $n \in \mathbb{N}$, all $x_1, \cdots, x_n \in \mathcal{X}$, and all $c_1, \cdots, c_n \in \mathbb{R}$, it holds that

$$\sum_{i,j=1}^{n} c_i c_j k(x_i, x_j) \geq 0 \tag{B}$$

It is called a *conditional positive definite kernel* if instead of for all $c_1, \cdots, c_n \in \mathbb{R}$ condition (B) only holds for all $c_1, \cdots, c_n \in \mathbb{R}$ with $\sum_{i=1}^n c_i = 0$.

Lemma A.1. Let $\mathcal{X}$ be a non-empty set, fix $x_0 \in \mathcal{X}$ and let $k, \tilde{k} : \mathcal{X} \times \mathcal{X} \to \mathbb{R}$ be symmetric functions satisfying for all $x, y \in \mathcal{X}$ that

$$k(x,y) = \tilde{k}(x,y) - \tilde{k}(x,x_0) - \tilde{k}(y,x_0) + \tilde{k}(x_0,x_0) \tag{C}$$

Then k is positive definite if and only if $\tilde{k}$ is conditionally positive definite.

Proof. Fix $n \in \mathbb{N}$, $c_1, \cdots, c_n \in \mathbb{R}$, and $x_0, x_1, \cdots, x_n \in \mathcal{X}$. Let $c_0 = -\sum_{i=1}^n c_i$, then we have

$$\begin{aligned}
\sum_{i,j=0}^{n} c_i c_j \tilde{k}(x_i,x_j) &= \sum_{i,j=1}^{n} c_i c_j \tilde{k}(x_i,x_j) + \sum_{i=1}^{n} c_i c_0 \tilde{k}(x_i,x_0) \\
&\quad + \sum_{j=1}^{n} c_0 c_j \tilde{k}(x_j,x_0) + c_0 c_0 \tilde{k}(x_0,x_0) \\
&= \sum_{i,j=1}^{n} c_i c_j \tilde{k}(x_i,x_j) - \sum_{i,j=1}^{n} c_i c_j \tilde{k}(x_i,x_0) \\
&\quad - \sum_{i,j=1}^{n} c_i c_j \tilde{k}(x_j,x_0) + \sum_{i,j=1}^{n} c_i c_j \tilde{k}(x_0,x_0) \\
&= \sum_{i,j=1}^{n} c_i c_j [\tilde{k}(x,y) - \tilde{k}(x,x_0) - \tilde{k}(y,x_0) + \tilde{k}(x_0,x_0)] \\
&= \sum_{i,j=1}^{n} c_i c_j k(x_i,x_j).
\end{aligned} \tag{D}$$

Now, if $\tilde{k}$ is conditionally positive definite, then (D) implies that $\sum_{i,j=1}^n c_i c_j k(x_i,x_j) \geq 0$, so k is positive definite; if k is positive definite, (D) implies that $\sum_{i,j=0}^n c_i c_j \tilde{k}(x_i, x_j \geq 0$ so $\tilde{k}$ is conditionally positive definite. This completes the proof of Lemma A.1. □

Lemma A.2 (Shifted conditionally positive definite)**.** Let $\mathcal{X}$ be a non-empty set and let $k : \mathcal{X} \times \mathcal{X} \to \mathbb{R}$ be a positive definite kernel, then

$$\tilde{k}(x,y) = k(x,y) + f(x) + f(y)$$

is a conditionally positive definite kernel for all $f : \mathcal{X} \to \mathbb{R}$.

Proof. The proof follows the exact same argument as the proof of Lemma A.1. □

Theorem A.2 (Connection between Hilbertian semi-metrics and positive definite kernels)**.** Let $k : \mathcal{X} \times \mathcal{X} \to \mathbb{R}$ and $d : \mathcal{X} \times \mathcal{X} \to [0, \infty)$ be functions. If k is a positive definite kernel and d satisfies $d^2(x, y) = k(x, x) + k(y, y) - 2k(x, y)$, then d is a Hilbertian semi-metric. On the other hand, for any $x_0 \in \mathcal{X}$, if d is a Hilbertian semi-metric and and k satisfies $k(x, y) = -\frac{1}{2}d^2(x, y) + \frac{1}{2}d^2(x, x_0) + \frac{1}{2}d^2(x_0, y)$, then k is a pd kernel.

The result is due to [2].

Proof. We start with the first part. Assume that k is a positive definite kernel and d satisfies $d^2(x, y) = k(x, x) + k(y, y) - 2k(x, y)$. Then, d is indeed a semi-metric by the following arguments:

(a) $d(x, x) = \sqrt{k(x, x) + k(x, x) - 2k(x, x)} = 0,$

(b) $d(x, y) = d(y, x)$, and since k is positive definite, let $c_1 = 1$, $c_2 = -1$, $x_1 = x$, and $x_2 = y$,

$$
\begin{aligned}
0 \le \sum_{i,j=1}^{n} c_i c_j k(x_i, x_j) &= k(x_1, x_1) - k(x_1, x_2) - k(x_2, x_1) + k(x_2, x_2) \\
&= k(x, x) + k(y, y) - 2k(x, y) \\
&= d(x, y)
\end{aligned}
$$

(c) Since k is a positive definite kernel, there exists a feature map ϕ_k from $\mathcal{X}$ to an RKHS $\mathcal{H}_k$, and we have

$$
\begin{aligned}
||\phi_k(x) - \phi_k(y)||^2_{\mathcal{H}_k} &= \langle \phi_k(x) - \phi_k(y), \phi_k(x) - \phi_k(y) \rangle_{\mathcal{H}_k} \\
&= \langle \phi_k(x), \phi_k(x) \rangle_{\mathcal{H}_k} + \langle \phi_k(y), \phi_k(y) \rangle_{\mathcal{H}_k} - 2\langle \phi_k(x), \phi_k(y) \rangle_{\mathcal{H}_k} \\
&= k(x, x) + k(y, y) - 2k(x, y) \\
&= d^2(x, y)
\end{aligned}
$$

Therefore, $d(x, z) \le d(x, y) + d(y, z)$ follows from the triangle inequality of a norm.

To show d is also Hilbertian, take any $n \in \mathbb{N}$, any $x_1, \cdots, x_n \in \mathcal{X}$, and any $c_1, \cdots, c_n \in \mathbb{R}$, we have

$$\begin{aligned}\sum_{i,j=1}^{n} c_i c_j d(x_i, x_j) &= \sum_{i=1}^{n} c_i k(x_i, x_i) \sum_{j=1}^{n} c_j + \sum_{j=1}^{n} c_i k(x_j, x_j) \sum_{i=1}^{n} c_i \\ &\quad - 2 \sum_{i,j=1}^{n} c_i c_j k(x_i, x_j) \\ &= -2 \sum_{i,j=1}^{n} c_i c_j k(x_i, x_j) \leq 0 \quad \text{(since } k \text{ is positive definite).}\end{aligned}$$

This proves the first part of the theorem.

For the second part, assume that d is a Hilbertian semi-metric and k satisfies $k(x, y) = -\frac{1}{2}d^2(x, y) + \frac{1}{2}d^2(x, x_0) + \frac{1}{2}d^2(x_0, y)$. Then, since d is Hilbertian, $-d^2$ satisfies the requirement of a conditionally positive definite kernel (with the additional property that $-d^2(x, x) = 0$). Hence, by Lemma A.1, k is indeed positive definite. This completes the proof of Theorem A.2.

□

B Dimensionality reduction and visualization with kernels

One important benefit of using the kernel approach is that we can leverage the kernels for dimensionality reduction and visualization, so that one can identify outliers in the data and further investigate them. In this section, we provide a short introduction on how to use kernels for multi-dimensional scaling and connect it to kernel PCA [3].

Kernel methods project the compositional data into a (potentially) high-dimensional RKHS $\mathcal{H}_k$, which we now want to project into the low dimensional Euclidean space $\mathbb{R}^\ell$ (with $\ell \ll p$) such that the lower dimensional representation preserves information that helps separate the observations of different traits in the RKHS. That is, given observations $x_1, \cdots, x_n \in \mathbb{S}^{p-1}$ and a kernel k, we would like to define a map $\Phi : \mathcal{H}_k \to \mathbb{R}^\ell$ such that

$$\sum_{i,j=1}^{n} \|\langle k(x_i, \cdot), k(x_j, \cdot)\rangle_{\mathcal{H}_k} - \langle \Phi(k(x_i, \cdot)), \Phi(k(x_j, \cdot))\rangle_{\mathbb{R}^\ell}\|^2$$

is minimized. In matrix notation, this corresponds to solving

$$\arg\min_{Z\in\mathbb{R}^{n\times\ell}} \|K - ZZ^\top\|^2,$$

where the rows of Z are $z_i = \Phi(k(x_i,\cdot)) \in \mathbb{R}^\ell$ for all $i \in \{1\cdots,n\}$ and $K \in \mathbb{R}^{n\times n}$ is the kernel Gram-matrix. This is similar to the classical multidimensional scaling (MDS) but measuring the similarity in the RKHS instead of in Euclidean space. By the Eckart-Young theorem [4], this minimization problem can be solved via the eigendecomposition of the matrix $K = V\Sigma V^\top$, and the optimal solution is

$$Z_{\text{opt}} = (V_1,\ldots,V_\ell)(\Sigma_{:\ell})^{\frac{1}{2}},$$

where $V_1,\ldots,V_\ell$ are the first ℓ columns of V and $\Sigma_{:\ell}$ is the upper-left $(\ell\times\ell)$-submatrix of Σ. The optimal projection Φ_{opt} is then given for all $f\in\mathcal{H}_k$ by

$$\Phi_{\text{opt}}(f) = (\Sigma_{:\ell})^{-\frac{1}{2}}(V_1,\ldots,V_\ell)^\top \begin{pmatrix} \langle f, k(x_1,\cdot)\rangle_{\mathcal{H}_k} \\ \vdots \\ \langle f, k(x_n,\cdot)\rangle_{\mathcal{H}_k} \end{pmatrix}. \tag{E}$$

This in particular allows to project a new observations $w \in \mathbb{S}^{p-1}$ with the same projection that is $w \mapsto \Phi_{\text{opt}}(k(w,\cdot))$.

The projection in (E) depends on the origin of the RKHS $\mathcal{H}_k$. To remove this dependence, it may therefore be desirable to consider a centered version of the optimal projection. This can be achieved by considering the RKHS $\tilde{\mathcal{H}}_k$ consisting of the functions $\tilde{f}(\cdot) = f(\cdot) - \frac{1}{n}\sum_{i=1}^n k(x_i,\cdot)$ with $f \in \mathcal{H}_k$. To compute the optimal centered projection (E) for the RKHS $\tilde{\mathcal{H}}_k$, we only need to perform double centering on the kernel matrix K, i.e., $\tilde{K} = HKH$, where $H = I - \frac{1}{n}\mathbf{1}\mathbf{1}^T$ and replace $k(x,\cdot)$ by $\tilde{k}(x,\cdot) = k(x,\cdot) - \frac{1}{n}\sum_{i=1}^n k(x_i,\cdot)$. With the centering step, this procedure is equivalent to kernel PCA [3]. The steps to obtain the lower-dimensional representation in matrix form are given in Algorithm 1.

Algorithm 1 Dimensionality reduction with kernels

Input: Training data $X_1, \ldots, X_n \in \mathbb{S}^{p-1}$, visualization data $X_1^{\text{new}}, \ldots, X_m^{\text{new}} \in \mathbb{S}^{p-1}$ (can be same as training data), kernel function k, dimension $l \in \{1, \ldots, p\}$, indicator whether to use centering CenterK $\in \{\texttt{True}, \texttt{False}\}$

Output: l-dimensional representation $Z = (Z_1, \ldots, Z_m)^\top \in \mathbb{R}^{m \times l}$

# Define centering function
function CenterKernelMatrix(K, $\tilde{K}$)
    $K^{\text{center}} \leftarrow \tilde{K} - \frac{1}{n}\mathbf{1}\mathbf{1}^T K - \frac{1}{n}\tilde{K}\mathbf{1}\mathbf{1}^T + \frac{1}{n^2}\mathbf{1}\mathbf{1}^T K \mathbf{1}\mathbf{1}^T$
    return K^{center}
end function

# Compute kernel matrix for training data
for $i, j = 1, \cdots, n$ **do**
    $K_{ij} \leftarrow k(X_i, X_j)$
end for

# Compute kernel matrix for visualization data
for $i = 1, \cdots, m$ and $j = 1, \cdots, n$ **do**
    $K_{ij}^{\text{new}} \leftarrow k(X_i^{\text{new}}, X_j)$
end for

# Center kernel matrices
if CenterK **then**
    $K^{\text{new}} \leftarrow \text{CenterKernelMatrix}(K, K^{\text{new}})$
    $K \leftarrow \text{CenterKernelMatrix}(K, K)$
end if

# Compute l-dimensional representation
$V, \Sigma \leftarrow$ eigenvalue decomposition of K
$Z \leftarrow K^{\text{new}}(V_1, \ldots, V_l)(\Sigma_{:l})^{-\frac{1}{2}}$

return Z

B.1 Compositionally adjusted coordinate-wise contribution to each principle component

Given the optimal projection function Φ_{opt}, define the function $F : \mathbb{S}^{p-1} \to \mathbb{R}^\ell$ for all $x \in \mathbb{S}^{p-1}$ by $F(x) = \Phi_{\text{opt}}(k(x, \cdot))$. We then call the components $F^1, \ldots, F^\ell$ the principle components. Our goal is now to understand how each principle component is affected by changes in the different components of its arguments. For this, fix a principle component $r \in \{1, \ldots, \ell\}$ and

consider for each $j \in \{1, \ldots, p\}$ the quantities

$$\mathbb{E}[F^r(\psi_j(X, c)) - F^r(X)],$$

where $c \in (0, 1)$ and ψ_j the perturbation defined in S1 Appendix. This is very similar in spirit as the CFI but with the derivative replaced by a difference and measures how much a perturbation of size c in the j-th component effects the value of the r-th principle component. It is easily estimated by

$$\frac{1}{n}\sum_{i=1}^{n} F^r(\psi_j(X_i, c)) - F^r(X_i).$$

References

1. Wells JH, Williams LR. Embeddings and extensions in analysis. vol. 84. Springer Science & Business Media; 2012.

2. Schoenberg IJ. Metric spaces and positive definite functions. Transactions of the American Mathematical Society. 1938;44.

3. Schölkopf B, Smola AJ, Bach F. Learning with kernels: support vector machines, regularization, optimization, and beyond. MIT press; 2002.

4. Eckart C, Young G. The approximation of one matrix by another of lower rank. Psychometrika. 1936;1(3):211–218.
