# Supplementary material for: Supervised learning and model analysis with compositional data
Source: PLoS Comput Biol. 2023 Jun 30;19(6):e1011240. doi: 10.1371/journal.pcbi.1011240 (PMC10343141; doi:10.1371/journal.pcbi.1011240)
Supplement: S6 Appendix — Proof of theorems and propositions. (PDF) [file pcbi.1011240.s006.pdf]

S6 Appendix: Proofs

A Proof of Proposition 2.1

Proof. We start with the CFI. Fix $j \in \{1, \ldots, p\}$ and $x \in \mathbb{S}^{p-1}$, then we can compute the derivative using the chain rule and the explicit form of the perturbation ψ as follows

$$\begin{aligned}
\tfrac{d}{dc} f(\psi_j(x,c)) &= \left\langle \nabla f(\psi_j(x,c)), \tfrac{d}{dc}\psi_j(x,c) \right\rangle \\
&= \left\langle \nabla f(\psi_j(x,c)), \tfrac{d}{dc} s_c(x^1, \cdots, x^{j-1}, cx^j, x^{j+1}, \cdots, x^p)^\top \right\rangle \\
&= \left\langle \nabla f(\psi_j(x,c)), \tfrac{d}{dc} \frac{1}{\sum_{\ell \neq j}^p x^\ell + cx^j} (x^1, \cdots, x^{j-1}, cx^j, x^{j+1}, \cdots, x^p)^\top \right\rangle \\
&= \Bigg\langle \nabla f(\psi_j(x,c)), \\
&\qquad \frac{-x^j}{(\sum_{\ell \neq j}^p x^\ell + cx^j)^2} \left(x^1, \cdots, x^{j-1}, cx^j x^j - x^j (\textstyle\sum_{\ell \neq j}^p x^\ell + cx^j), x^{j+1}, \cdots, x^p \right)^\top \Bigg\rangle.
\end{aligned}$$

Evaluating, the derivative at $c = 1$ leads to

$$\tfrac{d}{dc} f(\psi_j(x,c))|_{c=1} = \left\langle \nabla f(x), x^j(e_j - x) \right\rangle, \tag{A}$$

where we used that $\psi_j(x, 1) = x$. Moreover, the gradient of f in the case of the log-contrast model is given by

$$\nabla f(x) = \left(\frac{\beta_1}{x^1}, \ldots, \frac{\beta_p}{x^p} \right)^\top. \tag{B}$$

Combining (A) and (B) together with the constraint $\sum_{k=1}^p \beta_k = 0$ implies that

$$\tfrac{d}{dc} f(\psi_j(x,c))|_{c=1} = -x^j \sum_{k \neq j} \beta_k + \beta^j (1 - x^j) = \beta_j.$$

Hence, taking the expectation leads to

$$I_j^j = \mathbb{E}[\tfrac{d}{dc} f(\psi_j(X,c))|_{c=1}] = \beta_j,$$

which proves the first part of the proposition.

Next, we show the result for the CPD. Fix $j \in \{1,\ldots,p\}$ and $z \in [0,1]$. Then $S_f^j(z)$ for the log-contrast model can be computed as follows

$$\begin{aligned}
S_f^j(z) &= \mathbb{E}[f(\phi_j(X,z))] - \mathbb{E}[f(X)] \\
&= \sum_{\ell=1}^{p} \beta_\ell \mathbb{E}[\log(\phi_j(X,z)^\ell)] - \mathbb{E}[f(X)] \\
&= \sum_{\ell\neq j}^{p} \beta_\ell \mathbb{E}[\log(sX^\ell)] + \beta_j \log(z) - \mathbb{E}[f(X)] \\
&= \beta_j \log(z) + \sum_{\ell\neq j}^{p} \beta_\ell \mathbb{E}[\log(s)] + \sum_{\ell\neq j}^{p} \beta_\ell \mathbb{E}[\log(X^\ell)] - \mathbb{E}[f(X)],
\end{aligned}$$

where $s = (1-z)/(\sum_{\ell\neq j}^{p} X^\ell)$. Using $\beta^j = -\sum_{\ell\neq j}^{p} \beta_\ell$ (which follows from the log-contrast model constraint on β) we can simplify this further and get

$$\begin{aligned}
S_f^j(z) &= \beta_j \log(z) + \sum_{\ell\neq j}^{p} \beta_\ell \mathbb{E}[\log(1-z)] - \sum_{\ell\neq j}^{p} \beta_\ell \mathbb{E}[\log(\textstyle\sum_{k\neq j}^{p} X^k)] + \textstyle\sum_{\ell\neq j}^{p} \beta_\ell \mathbb{E}[\log(X^\ell)] - \mathbb{E}[f(X)] \\
&= \beta_j \log(z) - \beta_j \mathbb{E}[\log(1-z)] + \beta_j \mathbb{E}[\log(\textstyle\sum_{k\neq j}^{p} X^k)] + \textstyle\sum_{\ell\neq j}^{p} \beta_\ell \mathbb{E}[\log(X^\ell)] - \mathbb{E}[f(X)] \\
&= \beta_j \log\left(\frac{z}{1-z}\right) + \beta_j \mathbb{E}[\log(\textstyle\sum_{k\neq j}^{p} X^k)] + \textstyle\sum_{\ell\neq j}^{p} \beta_\ell \mathbb{E}[\log(X^\ell)] - \textstyle\sum_{\ell=1}^{p} \beta_\ell \mathbb{E}[\log(X^\ell)] \\
&= \beta_j \log\left(\frac{z}{1-z}\right) + c,
\end{aligned}$$

with $c = \beta_j \mathbb{E}[\log(\sum_{k\neq j}^{p} X^k)] + \sum_{\ell\neq j}^{p} \beta_\ell \mathbb{E}[\log(X^\ell)] - \sum_{\ell=1}^{p} \beta_\ell \mathbb{E}[\log(X^\ell)]$. Finally, assume $\beta^j = 0$, then it holds that

$$c = \sum_{\ell\neq j}^{p} \beta_\ell \mathbb{E}[\log(X^\ell)] - \sum_{\ell\neq j}^{p} \beta_\ell \mathbb{E}[\log(X^\ell)] = 0.$$

This completes the proof of Proposition 2.1. □

B Proof of Theorem 2.1

Proof. We first prove (i). To see this, we apply the triangle inequality to get that

$$|\hat{I}^j_{\hat{f}_n} - I^j_{f^*}| \leq \underbrace{|\hat{I}^j_{\hat{f}_n} - \hat{I}^j_{f^*}|}_{=:A_n} + \underbrace{|\hat{I}^j_{f^*} - I^j_{f^*}|}_{=:B_n}. \tag{C}$$

Next, we consider the two terms A_n and B_n separately. We begin with A_n, by using the definition of the CFI together with (A) from the proof of Proposition 2.1. This leads to

$$
\begin{aligned}
A_n &= \left| \frac{1}{n} \sum_{i=1}^{n} \left(\tfrac{d}{dc} \hat{f}_n(\psi(X_i, c)|_{c=1} - \tfrac{d}{dc} f^*(\psi(X_i, c)|_{c=1} \right) \right| \\
&= \left| \frac{1}{n} \sum_{i=1}^{n} \left\langle \nabla \hat{f}_n(X_i) - \nabla f^*(X_i), X_i^j(e_j - X_i) \right\rangle \right| \\
&\leq \frac{1}{n} \sum_{i=1}^{n} \left| \left\langle \nabla \hat{f}_n(X_i) - \nabla f^*(X_i), X_i^j(e_j - X_i) \right\rangle \right| \\
&\leq \frac{1}{n} \sum_{i=1}^{n} \left\| \nabla \hat{f}_n(X_i) - \nabla f^*(X_i) \right\|_2 \left\| X_i^j(e_j - X_i) \right\|_2 \\
&\leq \frac{1}{n} \sum_{i=1}^{n} \left\| \nabla \hat{f}_n(X_i) - \nabla f^*(X_i) \right\|_2,
\end{aligned}
$$

where for the last three steps we used the triangle inequality, the Cauchy-Schwartz inequality and that $\|X_i^j(e_j - X_i)\|_2 \leq 1$ since $X_i \in \mathbb{S}^{p-1}$, respectively. By assumption, it therefore holds that $A_n \to 0$ in probability as $n \to \infty$. For the B_n term, observe that using the same bounds it holds that

$$
\mathbb{E}\left[\left(\tfrac{d}{dc} f^*(\psi(X_i, c)|_{c=1} \right)^2 \right] = \mathbb{E}\left[\left(\left\langle \nabla f^*(X_i), X_i^j(e_j - X_i) \right\rangle \right)^2 \right] \leq \mathbb{E}\left[\|\nabla f^*(X_i)\|_2^2 \right].
$$

By assumption that $\mathbb{E}\left[\|\nabla f^*(X_i)\|_2^2 \right] < \infty$ this implies we can apply the weak law of large numbers to get for $n \to \infty$ that

$$
\hat{I}_{f^*}^j = \frac{1}{n} \sum_{i=1}^{n} \tfrac{d}{dc} f^*(\psi(X_i, c)|_{c=1} \xrightarrow{P} \mathbb{E}\left[\tfrac{d}{dc} f^*(\psi(X_i, c)|_{c=1} \right] = I_{f^*}^j.
$$

This immediately implies that $B_n \to 0$ in probability as $n \to \infty$. Combining the convergence of A_n and B_n in (C) completes the proof of (i).

Next, we prove (ii). Fix $j \in \{1, \ldots, p\}$ and $z \in [0, 1]$ such that $z/(1-z) \in \operatorname{supp}(X^j / \sum_{\ell \neq j} X^\ell)$. By the definition of the perturbation ϕ_j we get that

$$
\phi_j(X, z) = s(X^1, \cdots, X^{j-1}, \tfrac{z}{1-z} \textstyle\sum_{\ell \neq j} X^\ell, X^{j+1}, \cdots, X^p) \tag{D}
$$

where $s = (1-z)/(\sum_{\ell \neq j}^{p} X^{\ell})$. Next, using the assumption that $\mathrm{supp}(X) = \{x \in \mathbb{S}^{p-1} \mid x = w/(\sum_j w^j)$ with $w \in \mathrm{supp}(X^1) \times \cdots \times \mathrm{supp}(X^p)\}$ and that $z/(1-z) \in \mathrm{supp}(X^j/\sum_{\ell \neq j} X^{\ell})$ we get that

$$\phi_j(X, z) \in \mathrm{supp}(X^j) \tag{E}$$

almost surely.

By the triangle inequality it holds that

$$|\hat{S}^j_{\hat{f}_n}(z) - S^j_{f^*}(z)| \leq \underbrace{|\hat{S}^j_{\hat{f}_n}(z) - \hat{S}^j_{f^*}(z)|}_{=:C_n} + \underbrace{|\hat{S}^j_{f^*}(z) - S^j_{f^*}(z)|}_{=:D_n}. \tag{F}$$

We now consider the two terms C_n and D_n separately. First, we apply the triangle inequality to bound the C_n term as follows.

$$\begin{aligned}
C_n &= \left| \frac{1}{n} \sum_{i=1}^{n} (\hat{f}_n(\phi_j(X_i, z)) - f^*(\phi_j(X_i, z))) + \frac{1}{n} \sum_{i=1}^{n} (\hat{f}_n(X_i) - f^*(X_i)) \right| \\
&\leq \frac{1}{n} \sum_{i=1}^{n} \left| \hat{f}_n(\phi_j(X_i, z)) - f^*(\phi_j(X_i, z)) \right| + \frac{1}{n} \sum_{i=1}^{n} \left| \hat{f}_n(X_i) - f^*(X_i) \right| \\
&\leq 2 \sup_{x \in \mathrm{supp}(X)} \left| \hat{f}_n(x) - f^*(x) \right|,
\end{aligned}$$

where for the last step we used a supremum bound together with (D). Hence, using the assumption that $\sup_{x \in \mathrm{supp}(X)} |\hat{f}_n(x) - f^*(x)| \xrightarrow{P} 0$ as $n \to \infty$, we get that $C_n \to \infty$ in probability as $n \to \infty$. Similarly, for the D_n term we get that

$$\begin{aligned}
D_n &= \left| \frac{1}{n} \sum_{i=1}^{n} f^*(\phi_j(X_i, z)) - \mathbb{E}[f^*(\phi_j(X_i, z))] + \frac{1}{n} \sum_{i=1}^{n} f^*(X_i) - \mathbb{E}[f^*(X_i)] \right| \\
&\leq \left| \frac{1}{n} \sum_{i=1}^{n} f^*(\phi_j(X_i, z)) - \mathbb{E}[f^*(\phi_j(X_i, z))] \right| + \left| \frac{1}{n} \sum_{i=1}^{n} f^*(X_i) - \mathbb{E}[f^*(X_i)] \right|.
\end{aligned}$$

Since the $X_1, \ldots, X_n$ and hence $\phi_j(X_1, z), \ldots, \phi_j(X_n, z)$ are i.i.d. and bounded we can apply the weak law of large numbers to get that $D_n \to 0$ in probability as $n \to \infty$.

Finally, combining the convergence of C_n and D_n with (F) proves (ii) and hence completes the proof of Theorem 2.1. □

C Proof of Proposition 2.2

Proof. For this proof, we denote by $\mathbb{S}^{p-1}$ the open instead of the closed simplex.

First, since k_W is a positive definite kernel (see Section 2.1 in S2 Appendix for a proof), it holds that the RKHS $\mathcal{H}_{k_W}$ can be expressed as the closure of

$$\mathcal{F} := \left\{ f : \mathbb{S}^{p-1} \times \mathbb{S}^{p-1} \to \mathbb{R} \,\middle|\, \exists n \in \mathbb{N}, z_1, \dots, z_n \in \mathbb{S}^{p-1}, \alpha_1, \dots, \alpha_n \in \mathbb{R} : f(\cdot) = \textstyle\sum_{i=1}^n \alpha_i k_W(z_i, \cdot) \right\}.$$

We now show that any function in $\mathcal{F}$ has the expression given in the statement of the proposition. Let $f \in \mathcal{F}$ be arbitrary with the expansion

$$f(\cdot) = \sum_{i=1}^n \alpha_i k_W(z_i, \cdot).$$

Then, for all $x \in \mathbb{S}^{p-1}$ it holds that

$$\begin{aligned}
f(x) &= \sum_{i=1}^n \alpha_i \sum_{j,\ell=1}^p W_{\ell,j} \log\left(\frac{z_i^\ell}{g(z_i)}\right) \log\left(\frac{x^j}{g(x)}\right) \\
&= \sum_{j=1}^p \left(\sum_{\ell=1}^p W_{\ell,j} \sum_{i=1}^n \alpha_i \log\left(\frac{z_i^\ell}{g(z_i)}\right)\right) \log\left(\frac{x^j}{g(x)}\right) \\
&= \sum_{j=1}^p \left(\sum_{\ell=1}^p W_{\ell,j} \widetilde{\beta}_\ell \right) \log\left(\frac{x^j}{g(x)}\right) \\
&= \sum_{j=1}^p \left(\sum_{\ell=1}^p W_{\ell,j} \widetilde{\beta}_\ell \right) \log\left(x^j\right) - \left(\sum_{j,\ell=1}^p W_{\ell,j} \widetilde{\beta}_\ell \right) \log\left(g(x)\right) \\
&= \sum_{j=1}^p \left(\sum_{\ell=1}^p W_{\ell,j} \widetilde{\beta}_\ell \right) \log\left(x^j\right) - \left(\sum_{\ell=1}^p \widetilde{\beta}_\ell \right) \log\left(g(x)\right),
\end{aligned} \tag{G}$$

where in the third line we defined $\widetilde{\beta}_\ell := \sum_{i=1}^n \alpha_i \log\left(\frac{z_i^\ell}{g(z_i)}\right)$ and in the last equation we used that $\sum_{j=1}^p W_{\ell,j} = 1$ for all $\ell \in \{1, \dots, p\}$ by construction of W. Furthermore, we get that

$$\sum_{j=1}^p \widetilde{\beta}_j = \sum_{i=1}^n \alpha_i \left(\sum_{j=1}^p \log\left(z_i^j\right) - p \log(g(z_i)) \right) = \sum_{i=1}^n \alpha_i \left(\sum_{j=1}^p \log\left(z_i^j\right) - \sum_{j=1}^p \log\left(z_i^j\right) \right) = 0. \tag{H}$$

Now, combining this with (G) and setting $\beta_j := \sum_{\ell=1}^{p} W_{\ell,j}\widetilde{\beta}_\ell$ implies that

$$f(x) = \beta^\top \log(x),$$

where β does not depend on x.

It remains to show that β satisfies (i) $\sum_{j=1}^{p} \beta_j = 0$ and (ii) for all $\ell \in \{1, \ldots, m\}$ it holds for all $i, j \in P_\ell$ that $\beta_i = \beta_j$. For (i), we can use (H) and directly compute

$$\sum_{j=1}^{p} \beta_j = \sum_{j=1}^{p} \sum_{\ell=1}^{p} W_{\ell,j}\widetilde{\beta}_\ell = \sum_{\ell=1}^{p} \widetilde{\beta}_\ell = 0.$$

Finally for (ii), fix $k \in \{1, \ldots, m\}$ and $i, j \in P_k$, then it holds that

$$\beta_j = \sum_{\ell=1}^{p} W_{\ell,j}\widetilde{\beta}_\ell = \sum_{\ell=1}^{p} \sum_{r=1}^{m} \frac{1}{|P_r|} \mathbb{1}_{\{\ell,j \in P_r\}} \widetilde{\beta}_\ell = \sum_{\ell=1}^{p} \frac{1}{|P_k|} \widetilde{\beta}_\ell = \sum_{\ell=1}^{p} \sum_{r=1}^{m} \frac{1}{|P_r|} \mathbb{1}_{\{\ell,i \in P_r\}} \widetilde{\beta}_\ell = \sum_{\ell=1}^{p} W_{\ell,i}\widetilde{\beta}_\ell = \beta_i.$$

This completes the proof of Proposition 2.2. $\square$
