# Supplementary material for: Supervised learning and model analysis with compositional data
Source: PLoS Comput Biol. 2023 Jun 30;19(6):e1011240. doi: 10.1371/journal.pcbi.1011240 (PMC10343141; doi:10.1371/journal.pcbi.1011240)
Supplement: S7 Appendix — (PDF) [file pcbi.1011240.s007.pdf]

S7 Appendix: List of kernels implemented in `KernelBiome`

A List of unweighted kernels

In this section we summarize the all kernels implemented in `KernelBiome` and visualize the metrics and kernels via heatmaps when $p = 3$. The reference points are the neutral point $u = (\frac{1}{3}, \frac{1}{3}, \frac{1}{3})$, a vertex $v = (1, 0, 0)$, a midpoint on a boundary $m = (\frac{1}{2}, \frac{1}{2}, 0)$, and an interior point $z = (\frac{1}{4}, \frac{1}{4}, \frac{1}{2})$ of the simplex. For kernels we omit the neutral point, since $k(x, u) = 0$ for any $x \in \mathbb{S}^2$, as we centered our kernels at u.

Linear metric & kernel

$$d^2(x, y) = \sum_{j=1}^{p} (x^j - y^j)^2$$

$$k(x, y) = \left(\sum_{j=1}^{p} x^j y^j \right) - \frac{1}{p}$$

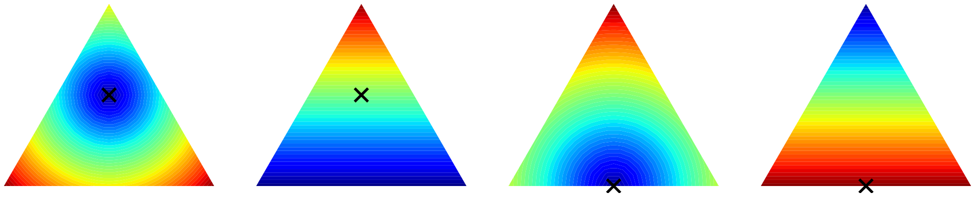

$d(x, z)$ $k(x, z)$ $d(x, m)$ $k(x, m)$

RBF metric & kernel

$$d^2(x,y) = 2 - 2\exp\Big(\sum_{j=1}^{p}\frac{(x^j-y^j)^2}{2\sigma^2}\Big)$$

$$k(x,y) = \exp\Big(-\sum_{j=1}^{p}\frac{(x^j-y^j)^2}{2\sigma^2}\Big)$$

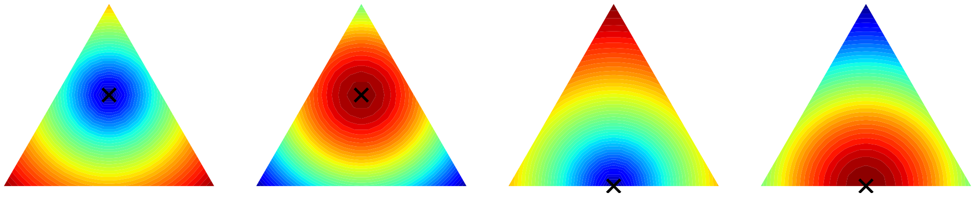

$d_{\sigma=\frac{1}{\sqrt{2}}}(x,z)$ $k_{\sigma=\frac{1}{\sqrt{2}}}(x,z)$ $d_{\sigma=\frac{1}{\sqrt{2}}}(x,m)$ $k_{\sigma=\frac{1}{\sqrt{2}}}(x,m)$

Generalized-JS metric & kernel $(a<\infty, b\in[0.5,a))$

$$d^2(x,y) = \frac{ab}{a-b}\sum_{j=1}^{p}\frac{2^{\frac{1}{b}}\Big[(x^j)^a+(y^j)^a\Big]^{\frac{1}{a}} - 2^{\frac{1}{a}}\Big[(x^j)^b+(y^j)^b\Big]^{\frac{1}{b}}}{2^{\frac{1}{a}+\frac{1}{b}}}$$

$$\begin{aligned}k(x,y) = -\frac{ab}{a-b}\cdot 2^{-(1+\frac{1}{a}+\frac{1}{b})}\sum_{j=1}^{p}\Bigg\{&2^{\frac{1}{b}}\Big(\Big[(x^j)^a+(y^j)^a\Big]^{\frac{1}{a}} - \Big[(x^j)^a+(\tfrac{1}{p})^a\Big]^{\frac{1}{a}}\\ &-\Big[(\tfrac{1}{p})^a+(y^j)^a\Big]^{\frac{1}{a}}\Big) - 2^{\frac{1}{a}}\Big(\Big[(x^j)^b+(y^j)^b\Big]^{\frac{1}{b}} - \Big[(x^j)^b+(\tfrac{1}{p})^b\Big]^{\frac{1}{b}}\\ &-\Big[(\tfrac{1}{p})^b+(y^j)^b\Big]^{\frac{1}{b}}\Big)\Bigg\}\end{aligned}$$

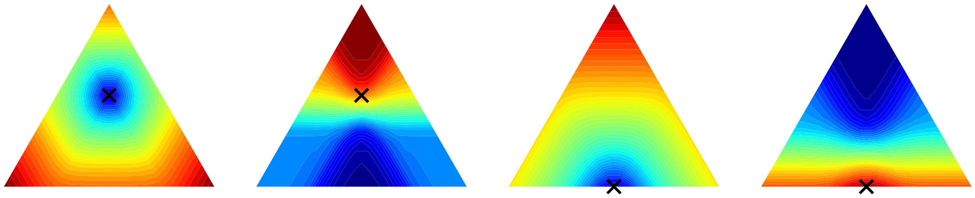

$d_{a=10,b=1}(x,z)$ $k_{a=10,b=1}(x,z)$ $d_{a=10,b=1}(x,z)$ $k_{a=10,b=1}(x,z)$

Generalized-JS metric & kernel $(a \to \infty, b < \infty)$

$$d^2(x,y) = \sum_{j=1}^{p} b \left\{ 2^{\frac{1}{b}} \cdot \max\{x^j, y^j\} - \left[(x^j)^b + (y^j)^b \right]^{\frac{1}{b}} \right\}$$

$$k(x,y) = -\frac{b}{2} \sum_{j=1}^{p} \left\{ 2^{\frac{1}{b}} \left(\max\{x^j, y^j\} - \max\{x^j, \tfrac{1}{p}\} - \max\{y^j, \tfrac{1}{p}\} \right) \right.$$
$$\left. - \left[(x^j)^b + (y^j)^b \right]^{\frac{1}{b}} + \left[(x^j)^b + (\tfrac{1}{p})^b \right]^{\frac{1}{b}} + \left[(y^j)^b + (\tfrac{1}{p})^b \right]^{\frac{1}{b}} \right\}$$

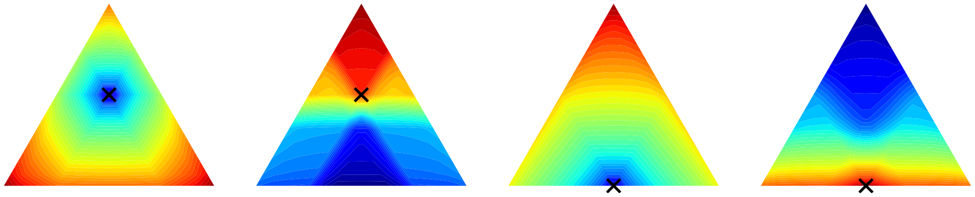

$d_{a=\infty,b=0.5}(x,z)$ $k_{a=\infty,b=0.5}(x,z)$ $d_{a=\infty,b=0.5}(x,m)$ $k_{a=\infty,b=0.5}(x,m)$

Generalized-JS metric & kernel $(a < \infty, b \to a)$

$$d^2(x,y) = \sum_{j=1}^{p} \left[\frac{(x^j)^b + (y^j)^b}{2}\right]^{\frac{1}{b}} \cdot \left[\frac{(x^j)^b}{(x^j)^b + (y^j)^b} \cdot \log \frac{2(x^j)^b}{(x^j)^b + (y^j)^b} + \frac{(y^j)^b}{(x^j)^b + (y^j)^b} \cdot \log \frac{2(y^j)^b}{(x^j)^b + (y^j)^b}\right]$$

$$k(x,y) = -\frac{1}{2^{\frac{1}{b}+1}} \sum_{j=1}^{p} \Bigg\{ \left[(x^j)^b + (y^j)^b\right]^{\frac{1}{b}-1} \cdot \Big((x^j)^b \cdot \log\left[2(x^j)^b\right] + (y^j)^b \log\left[2(y^j)^b\right] - \left[(x^j)^b + (y^j)^b\right] \cdot \log\left[(x^j)^b + (y^j)^b\right] \Big)$$
$$- \left[(x^j)^b + \tfrac{1}{p^b}\right]^{\frac{1}{b}-1} \cdot \Big(- \left[(x^j)^b + (\tfrac{1}{p^b})\right] \cdot \log\left[(x^j)^b + \tfrac{1}{p^b}\right] + (x^j)^b \cdot \log\left[2(x^j)^b\right] + \tfrac{1}{p^b} \cdot \log\left[\tfrac{2}{p^b}\right] \Big)$$
$$- \left[(y^j)^b + \tfrac{1}{p^b}\right]^{\frac{1}{b}-1} \cdot \Big(- \left[(y^j)^b + (\tfrac{1}{p^b})\right] \cdot \log\left[(y^j)^b + \tfrac{1}{p^b}\right] + (y^j)^b \cdot \log\left[2(y^j)^b\right] + \tfrac{1}{p^b} \cdot \log\left[\tfrac{2}{p^b}\right] \Big) \Bigg\}$$

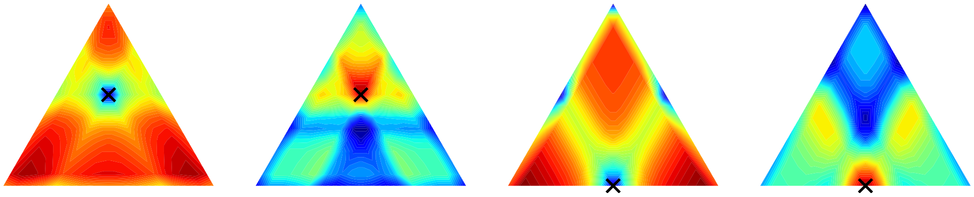

$d_{a=b=10}(x,z)$ $k_{a=b=10}(x,z)$ $d_{a=b=10}(x,m)$ $k_{a=b=10}(x,m)$

Generalized-JS metric & kernel ($a = b \to \infty$)

$$d^2(x, y) = \sum_{j=1}^{p} \max\{x^j, y^j\} \cdot \Big[\log(2)\mathbb{1}\{x^j \neq y^j\}\Big]$$

$$k(x, y) = -\frac{\log(2)}{2} \cdot \sum_{j=1}^{p} \Big\{ \max\{x^j, y^j\} \cdot \mathbb{1}\{x^j \neq y^j\}$$
$$- \max\{x^j, \tfrac{1}{p}\} \cdot \mathbb{1}\{x^j \neq \tfrac{1}{p}\} - \max\{y^j, \tfrac{1}{p}\} \cdot \mathbb{1}\{y^j \neq \tfrac{1}{p}\} \Big\}$$

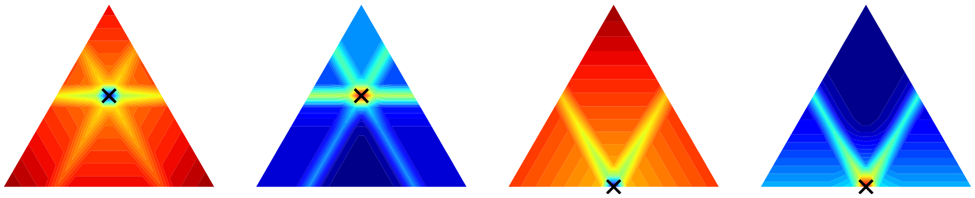

$d_{a=b=\infty}(x, z)$ $k_{a=b=\infty}(x, z)$ $d_{a=b=\infty}(x, m)$ $k_{a=b=\infty}(x, m)$

Special Case: Hellinger - Generalized-JS metric & kernel ($a = 1, b = \frac{1}{2}$)

$$d^2(x, y) = \frac{\sqrt{2}}{2} \sum_{j=1}^{p} \left(\sqrt{x^j} - \sqrt{y^j}\right)^2$$

$$k(x, y) = \frac{\sqrt{2}}{4} + \frac{\sqrt{2}}{4} \sum_{j=1}^{p} \left\{ \sqrt{x^j y^j} - \frac{\sqrt{x^j} + \sqrt{y^j}}{\sqrt{p}} \right\}$$

This corresponds to $\frac{\sqrt{2}}{2}$ times the **Hellinger** metric and kernel.

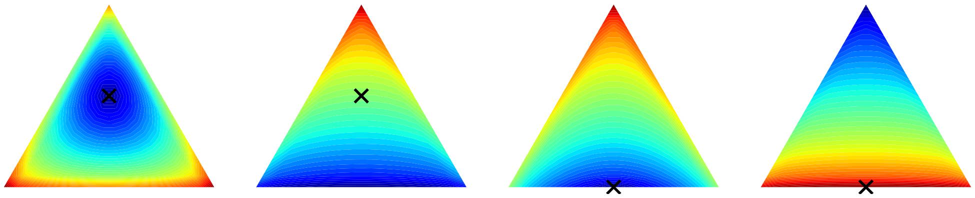

$d_{a=1,b=0.5}(x, z)$ $k_{a=1,b=0.5}(x, z)$ $d_{a=1,b=0.5}(x, m)$ $k_{a=1,b=0.5}(x, m)$

Special Case: Jenson-Shannon - Generalized-JS metric & kernel $(a=1, b=1)$

$$d^2(x,y) = \frac{1}{2}\sum_{j=1}^{p} x^j \log\frac{2x^j}{x^j+y^j} + y^j \log\frac{2y^j}{x^j+y^j}$$

$$k(x,y) = -\frac{1}{4}\sum_{j=1}^{p}\left\{x^j \log\frac{x^j+\frac{1}{p}}{x^j+y^j} + y^j \log\frac{y^j+\frac{1}{p}}{x^j+y^j} - \frac{1}{p}\log\frac{4}{p^2(x^j+\frac{1}{p})(y^j+\frac{1}{p})}\right\}$$

This corresponds to the **Jenson-Shannon** metric and kernel.

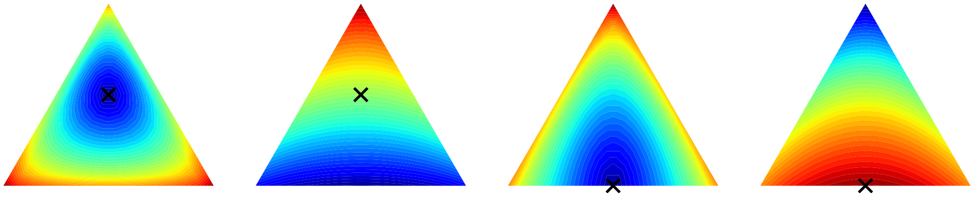

$d_{a=b=1}(x,z)$ $k_{a=b=1}(x,z)$ $d_{a=b=1}(x,z)$ $k_{a=b=1}(x,z)$

Special Case: Total Variation - Generalized-JS metric & kernel $(a=\infty, b=1)$

$$d^2(x,y) = \sum_{j=1}^{p} |x^j - y^j|$$

$$k(x,y) = -\frac{1}{2}\sum_{j=1}^{p}\left\{|x^j - y^j| - |x^j - \tfrac{1}{p}| - |y^j - \tfrac{1}{p}|\right\}$$

This corresponds to 2 times the **total variation** metric and kernel.

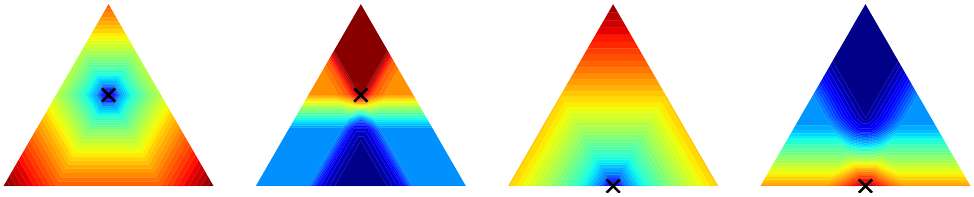

$d_{a=\infty,b=1}(x,z)$ $k_{a=\infty,b=1}(x,z)$ $d_{a=\infty,b=1}(x,z)$ $k_{a=\infty,b=1}(x,z)$

Hilbertian metric & kernel $(a < \infty, b > -\infty)$

$$d^2(x,y) = \sum_{j=1}^{p} \frac{2^{\frac{1}{b}} \left[(x^j)^a + (y^j)^a \right]^{\frac{1}{a}} - 2^{\frac{1}{a}} \left[(x^j)^b + (y^j)^b \right]^{\frac{1}{b}}}{2^{\frac{1}{a}} - 2^{\frac{1}{b}}}$$

$$\begin{aligned} k(x,y) = -\frac{1}{2(2^{\frac{1}{a}} - 2^{\frac{1}{b}})} \sum_{j=1}^{p} \Bigg\{ & 2^{\frac{1}{b}} \Big(\left[(x^j)^a + (y^j)^a \right]^{\frac{1}{a}} - \left[(x^j)^a + (\tfrac{1}{p})^a \right]^{\frac{1}{a}} \\ & - \left[(y^j)^a + (\tfrac{1}{p})^a \right]^{\frac{1}{a}} \Big) - 2^{\frac{1}{a}} \Big(\left[(x^j)^b + (y^j)^b \right]^{\frac{1}{b}} - \left[(x^j)^b + (\tfrac{1}{p})^b \right]^{\frac{1}{b}} \\ & - \left[(y^j)^b + (\tfrac{1}{p})^b \right]^{\frac{1}{b}} \Big) \Bigg\} \end{aligned}$$

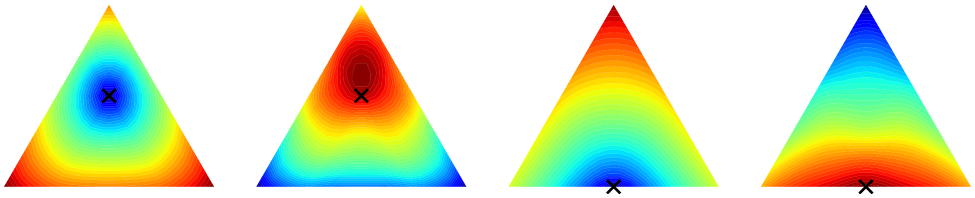

$d_{a=10,b=-1}(x,z)$ $k_{a=10,b=-1}(x,z)$ $d_{a=10,b=-1}(x,m)$ $k_{a=10,b=-1}(x,m)$

Hilbertian metric & kernel $(a \to \infty, b > -\infty)$

$$d^2(x,y) = \sum_{j=1}^{p} b \left\{ 2^{\frac{1}{b}} \cdot \max\{x^j, y^j\} - \left[(x^j)^b + (y^j)^b \right]^{\frac{1}{b}} \right\}$$

$$\begin{aligned} k(x,y) = -\frac{1}{2(1 - 2^{\frac{1}{b}})} \sum_{j=1}^{p} \Bigg\{ & 2^{\frac{1}{b}} \Big(\max\{x^j, y^j\} - \max\{x^j, \tfrac{1}{p}\} - \max\{y^j, \tfrac{1}{p}\} \Big) \\ & - \left[(x^j)^b + (y^j)^b \right]^{\frac{1}{b}} + \left[(x^j)^b + (\tfrac{1}{p})^b \right]^{\frac{1}{b}} + \left[(y^j)^b + (\tfrac{1}{p})^b \right]^{\frac{1}{\beta}} \Bigg\} \end{aligned}$$

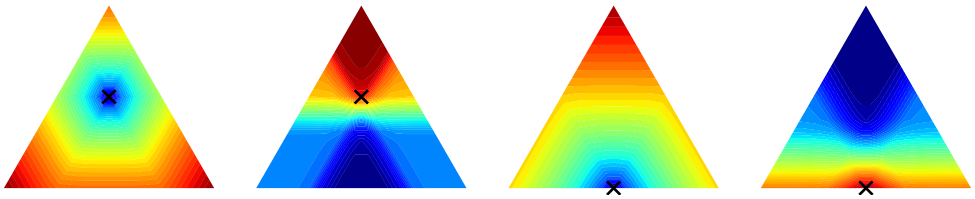

$d_{a=\infty,b=-10}(x,z)$ $k_{a=\infty,b=-10}(x,z)$ $d_{a=\infty,b=-10}(x,m)$ $k_{a=\infty,b=-10}(x,m)$

Hilbertian metric & kernel $(a < \infty, b \to -\infty)$

$$d^2(x,y) = \frac{1}{2^{\frac{1}{a}} - 1} \sum_{j=1}^{p} \left\{ \left[(x^j)^a + (y^j)^a \right]^{\frac{1}{a}} - 2^{\frac{1}{a}} \cdot \min\{x^j, y^j\} \right\}$$

$$k(x,y) = -\frac{1}{2(2^{\frac{1}{a}} - 1)} \sum_{j=1}^{p} \left\{ \left[(x^j)^a + (y^j)^a \right]^{\frac{1}{a}} - \left[(x^j)^a + (\tfrac{1}{p})^a \right]^{\frac{1}{a}} - \left[(y^j)^a + (\tfrac{1}{p})^a \right]^{\frac{1}{a}} - 2^{\frac{1}{a}} \left[\min\{x^j, y^j\} - \min\{x^j, \tfrac{1}{p}\} - \min\{y^j, \tfrac{1}{p}\} \right] \right\}$$

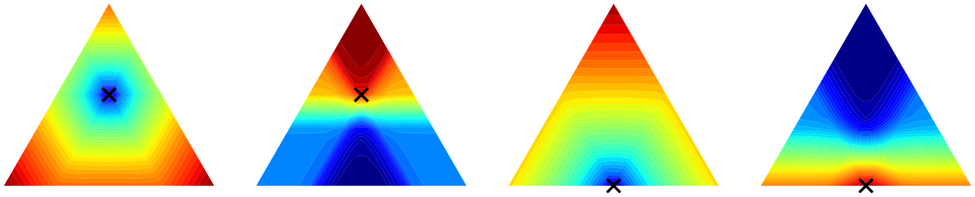

$d_{a=10,b=-\infty}(x,z)$ $k_{a=10,b=-\infty}(x,z)$ $d_{a=10,b=-\infty}(x,m)$ $k_{a=10,b=-\infty}(x,m)$

Special Case: Chi-square - Hilbertian metric & kernel $(a = 1, b = -1)$

$$d^2(x,y) = \frac{1}{3} \sum_{j=1}^{p} \frac{(x^j - y^j)^2}{x^j + y^j}$$

$$k(x,y) = -\frac{1}{6} \sum_{j=1}^{p} \left\{ \frac{(x^j - y^j)^2}{x^j + y^j} - \frac{(x^j - \frac{1}{p})^2}{x^j + \frac{1}{p}} - \frac{(y^j - \frac{1}{p})^2}{y^j + \frac{1}{p}} \right\}$$

This corresponds to $\frac{1}{3}$ of the **chi-square** metric and kernel.

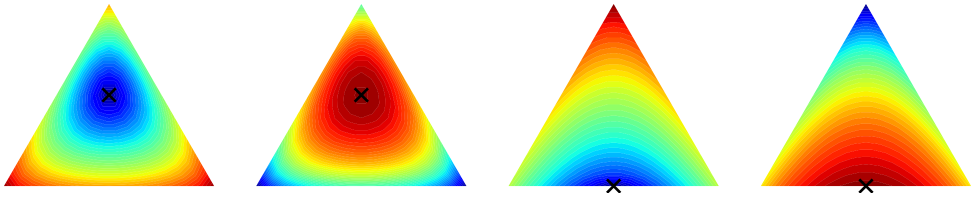

$d_{a=1,b=-1}(x,z)$ $k_{a=1,b=-1}(x,z)$ $d_{a=1,b=-1}(x,m)$ $k_{a=1,b=-1}(x,m)$

Special Case: Total Variation - Hilbertian metric & kernel $(a = 1, b = -\infty)$

$$d^2(x, y) = \frac{1}{2}\sum_{j=1}^{p} |x^j - y^j|$$

$$k(x, y) = -\frac{1}{4}\sum_{j=1}^{p}\left\{|x^j - y^j| - |x^j - \tfrac{1}{p}| - |y^j - \tfrac{1}{p}|\right\}$$

This corresponds to the **total variation** metric and kernel.

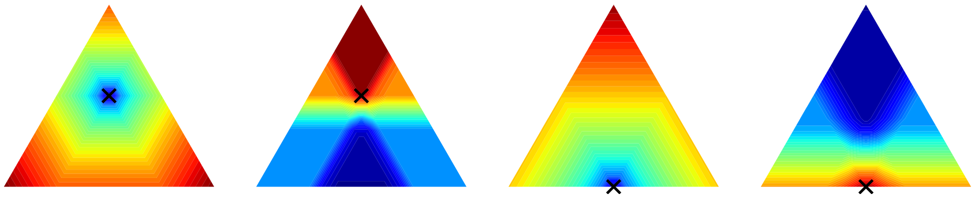

$d_{a=1,b=-\infty}(x, z)$ $k_{a=1,b=-\infty}(x, z)$ $d_{a=1,b=-\infty}(x, m)$ $k_{a=1,b=-\infty}(x, m)$

Aitchison metric & kernel

$$d^2(x, y) = \sum_{j=1}^{p}\left(\log\frac{x^j + c}{g(x + c)} - \log\frac{y^j + c}{g(y + c)}\right)^2$$

$$k(x, y) = \sum_{j=1}^{p}\log\frac{x^j + c}{g(x + c)}\log\frac{y^j + c}{g(y + c)}$$

where $g(x) = \sqrt[p]{\prod_{j=1}^{p} x^j}$ is the geometric mean of x.

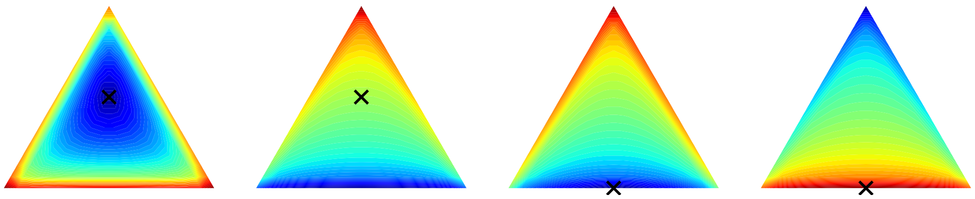

$d_{c=0.01}(x, z)$ $k_{c=0.01}(x, z)$ $d_{c=0.01}(x, m)$ $k_{c=0.01}(x, m)$

Aitchison-RBF metric & kernel

$$d^2(x,y) = 2 - 2\exp\Big(-\frac{1}{2\sigma^2}\sum_{j=1}^{p}\Big[\log\frac{x^j+c}{g(x+c)} - \log\frac{y^j+c}{g(y+c)}\Big]^2\Big)$$

$$k(x,y) = \exp\Big(-\frac{1}{2\sigma^2}\sum_{j=1}^{p}\Big[\log\frac{x^j+c}{g(x+c)} - \log\frac{y^j+c}{g(y+c)}\Big]^2\Big)$$

where $g(x) = \sqrt[p]{\prod_{j=1}^{p} x^j}$ is the geometric mean of x.

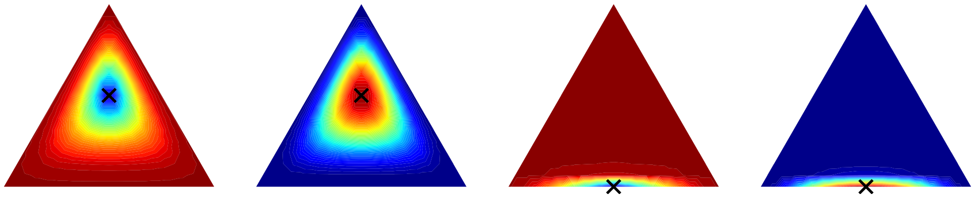

$d_{\sigma=\frac{1}{\sqrt{2}},c=0.01}(x,z)$ $k_{\sigma=\frac{1}{\sqrt{2}},c=0.01}(x,z)$ $d_{\sigma=\frac{1}{\sqrt{2}},c=0.01}(x,m)$ $k_{\sigma=\frac{1}{\sqrt{2}},c=0.01}(x,m)$

Heat diffusion metric & kernel

$$d^2(x,y) = 2\cdot(4\pi t)^{-\frac{p}{2}}\cdot\Big[1-\exp\Big(-\tfrac{1}{t}\arccos^2(\sum_{j=1}^{p}\sqrt{x^j y^j})\Big)\Big]$$

$$k(x,y) = (4\pi t)^{-p/2}\cdot\exp\Big(-\tfrac{1}{t}\arccos^2(\sum_{j=1}^{p}\sqrt{x^j y^j})\Big)$$

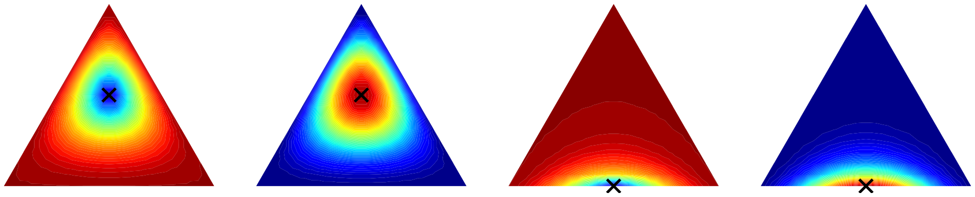

$d_{t=0.1}(x,z)$ $k_{t=0.1}(x,z)$ $d_{t=0.1}(x,m)$ $k_{t=0.1}(x,m)$

B List of weighted kernels

As discussed in S2 Appendix, all kernels can also be modified to include a weight matrix $W \in \mathbb{R}^{p\times p}$. Below, we list the explicit forms of all weighted kernels as they are implemented in `KernelBiome` package. As before, let $g(x) = \sqrt[p]{\prod_{j=1}^{p} x^j}$ be the geometric mean of x.

Linear kernel

$$k(x,y) = \sum_{j,\ell=1}^{p} W_{j,\ell}\Big(x^j y^\ell - \tfrac{x^j}{p} - \tfrac{y^\ell}{p} + \tfrac{1}{p^2}\Big)$$

RBF kernel

$$k(x,y) = \exp\Big(- \sum_{j,\ell=1}^{p} \frac{W_{j,\ell}(x^j - y^\ell)^2}{2\sigma^2}\Big)$$

Generalized-JS kernel ($a < \infty, b \in [0.5, a]$)

$$\begin{aligned} k(x,y) = -\frac{ab}{a-b} \cdot 2^{-(1+\frac{1}{a}+\frac{1}{b})} \sum_{j,\ell=1}^{p} W_{j,\ell}\Bigg\{ & 2^{\frac{1}{b}}\Big(\Big[(x^j)^a + (y^\ell)^a\Big]^{\frac{1}{a}} - \Big[(x^j)^a + (\tfrac{1}{p})^a\Big]^{\frac{1}{a}} \\ & - \Big[(\tfrac{1}{p})^a + (y^\ell)^a\Big]^{\frac{1}{a}}\Big) - 2^{\frac{1}{a}}\Big(\Big[(x^j)^b + (y^\ell)^b\Big]^{\frac{1}{b}} - \Big[(x^j)^b + (\tfrac{1}{p})^b\Big]^{\frac{1}{b}} \\ & - \Big[(\tfrac{1}{p})^b + (y^\ell)^b\Big]^{\frac{1}{b}}\Big)\Bigg\} \end{aligned}$$

Generalized-JS kernel ($a \to \infty, b < \infty$)

$$\begin{aligned} k(x,y) = -\frac{b}{2} \sum_{j,\ell=1}^{p} W_{j,\ell}\Bigg\{ & 2^{\frac{1}{b}}\Big(\max\{x^j, y^\ell\} - \max\{x^j, \tfrac{1}{p}\} - \max\{y^\ell, \tfrac{1}{p}\}\Big) \\ & - \Big[(x^j)^b + (y^\ell)^b\Big]^{\frac{1}{b}} + \Big[(x^j)^b + (\tfrac{1}{p})^b\Big]^{\frac{1}{b}} + \Big[(y^\ell)^b + (\tfrac{1}{p})^b\Big]^{\frac{1}{b}}\Bigg\} \end{aligned}$$

Generalized-JS kernel ($a < \infty, b \to a$)

$$\begin{aligned} k(x,y) = -\frac{1}{2^{\frac{1}{b}+1}} \sum_{j,\ell=1}^{p} W_{j,\ell}\Bigg\{ & \Big[(x^j)^b + (y^\ell)^b\Big]^{\frac{1}{b}-1} \cdot \Big((x^j)^b \cdot \log\Big[2(x^j)^b\Big] + (y^\ell)^b \log\Big[2(y^\ell)^b\Big] \\ & - \Big[(x^j)^b + (y^\ell)^b\Big] \cdot \log\Big[(x^j)^b + (y^\ell)^b\Big]\Big) \\ & - \Big[(x^j)^b + \tfrac{1}{p^b}\Big]^{\frac{1}{b}-1} \cdot \Big(- \Big[(x^j)^b + (\tfrac{1}{p^b})\Big] \cdot \log\Big[(x^j)^b + \tfrac{1}{p^b}\Big] \\ & + (x^j)^b \cdot \log\Big[2(x^j)^b\Big] + \tfrac{1}{p^b} \cdot \log\Big[\tfrac{2}{p^b}\Big]\Big) \\ & - \Big[(y^\ell)^b + \tfrac{1}{p^b}\Big]^{\frac{1}{b}-1} \cdot \Big(- \Big[(y^\ell)^b + (\tfrac{1}{p^b})\Big] \cdot \log\Big[(y^\ell)^b + \tfrac{1}{p^b}\Big] \\ & + (y^\ell)^b \cdot \log\Big[2(y^\ell)^b\Big] + \tfrac{1}{p^b} \cdot \log\Big[\tfrac{2}{p^b}\Big]\Big)\Bigg\} \end{aligned}$$

Generalized-JS kernel ($a = b \to \infty$)

$$k(x,y) = -\frac{\log(2)}{2} \cdot \sum_{j,\ell=1}^{p} W_{j,\ell} \Bigg\{ \max\{x^j, y^\ell\} \cdot \mathbb{1}\{x^j \neq y^\ell\} - \max\{x^j, \tfrac{1}{p}\} \cdot \mathbb{1}\{x^j \neq \tfrac{1}{p}\} - \max\{y^\ell, \tfrac{1}{p}\} \cdot \mathbb{1}\{y^\ell \neq \tfrac{1}{p}\} \Bigg\}$$

Hilbertian kernel ($a < \infty, b > -\infty$)

$$k(x,y) = -\frac{1}{2(2^{\frac{1}{a}} - 2^{\frac{1}{b}})} \sum_{j,\ell=1}^{p} W_{j,\ell} \Bigg\{ 2^{\frac{1}{b}} \Big(\Big[(x^j)^a + (y^\ell)^a\Big]^{\frac{1}{a}} - \Big[(x^j)^a + (\tfrac{1}{p})^a\Big]^{\frac{1}{a}} - \Big[(y^\ell)^a + (\tfrac{1}{p})^a\Big]^{\frac{1}{a}} \Big) - 2^{\frac{1}{a}} \Big(\Big[(x^j)^b + (y^\ell)^b\Big]^{\frac{1}{b}} - \Big[(x^j)^b + (\tfrac{1}{p})^b\Big]^{\frac{1}{b}} - \Big[(y^\ell)^b + (\tfrac{1}{p})^b\Big]^{\frac{1}{b}} \Big) \Bigg\}$$

Hilbertian kernel ($a \to \infty, b > -\infty$)

$$k(x,y) = -\frac{1}{2(1 - 2^{\frac{1}{b}})} \sum_{j,\ell=1}^{p} W_{j,\ell} \Bigg\{ 2^{\frac{1}{b}} \Big(\max\{x^j, y^\ell\} - \max\{x^j, \tfrac{1}{p}\} - \max\{y^\ell, \tfrac{1}{p}\} \Big) - \Big[(x^j)^b + (y^\ell)^b\Big]^{\frac{1}{b}} + \Big[(x^j)^b + (\tfrac{1}{p})^b\Big]^{\frac{1}{b}} + \Big[(y^\ell)^b + (\tfrac{1}{p})^b\Big]^{\frac{1}{b}} \Bigg\}$$

Hilbertian kernel ($a < \infty, b \to -\infty$)

$$k(x,y) = -\frac{1}{2(2^{\frac{1}{a}} - 1)} \sum_{j,\ell=1}^{p} W_{j,\ell} \Bigg\{ \Big[(x^j)^a + (y^\ell)^a\Big]^{\frac{1}{a}} - \Big[(x^j)^a + (\tfrac{1}{p})^a\Big]^{\frac{1}{a}} - \Big[(y^\ell)^a + (\tfrac{1}{p})^a\Big]^{\frac{1}{a}} - 2^{\frac{1}{a}} \Big[\min\{x^j, y^\ell\} - \min\{x^j, \tfrac{1}{p}\} - \min\{y^\ell, \tfrac{1}{p}\} \Big] \Bigg\}$$

Aitchison kernel

$$k(x,y) = \sum_{j,\ell=1}^{p} W_{j,\ell} \log \frac{x^j + c}{g(x+c)} \log \frac{y^\ell + c}{g(y+c)}$$

Aitchison RBF kernel

$$k(x,y) = \exp\Big(-\frac{1}{2\sigma^2} \sum_{j,\ell=1}^{p} W_{j,\ell} \Big[\log \frac{x^j + c}{g(x+c)} - \log \frac{y^\ell + c}{g(y+c)} \Big]^2 \Big)$$

Heat diffusion kernel

$$k(x,y) = (4\pi t)^{-p/2} \cdot \exp\left(-\frac{1}{t} \arccos^2\left(\sum_{j,\ell=1}^{p} W_{j,\ell} \sqrt{x^j y^\ell} \right) \right)$$
