# Supplementary material for: Supervised learning and model analysis with compositional data
Source: PLoS Comput Biol. 2023 Jun 30;19(6):e1011240. doi: 10.1371/journal.pcbi.1011240 (PMC10343141; doi:10.1371/journal.pcbi.1011240)
Supplement: Attachment — Submitted filename: response_to_reviewers.pdf [file pcbi.1011240.s008.pdf]

Point-by-point response to the reviews' comments on
"Supervised Learning and Model Analysis with Compositional Data"

We thank the reviewers and the members of the editorial board for their feedback. We have revised the manuscript according to the feedback and believe that this has improved its quality. The major changes in this revision include the following points:

- Created a summary figure for the evaluation on all 33 microbiome sets.
- Extended the prediction experiment as follows: (1) included a Wilcoxon signed-rank test to check which methods are significantly outperformed by competitors, (2) changed metrics to balanced accurary, RMSE and precision-recall curves, and (3) described time complexity and demonstrated empirical run times.
- Added a new semi-synthetic experiment illustrating when prior information is useful and harmful in terms of prediction.

Response to members of the editorial board

The reviewers both agree on the value of the study, an opinion we share, but request additional clarifications. We note that a common theme is both reviewers wish to see a clearer statement of how the proposed methods compared to others, which we agree is important. As an additional editorial request, we ask that the authors rework the figures to avoid the use of very small fonts (particularly Fig 1 [bottom right] and Fig 4).

Response: Thank you for handling our manuscript. In the revision we have substantially updated the experimental section according to the reviewers' comments.

Additionally, we have reworked all Figures to increase the font size. Please let us know if any of the fonts are still to small.

Response to Reviewer #1

Summary:

The manuscript by Shimeng Huang et al. proposes KernelBiome, a kernel-based nonparametric regression and classification framework for compositional data. The method is specifically developed to deal with sparse compositional data and can incorporate prior knowledge in terms of phylogenetic structure.

The algorithm is validated experimentally on 33 publicly available microbiome datasets and compared with state-of-the-art solutions. The code is available as an open-source python package. The topic involved in the paper is suitable for publication in PLOS Computational Biology. I find the methodological solution quite interesting. It is described in detail in both the main paper and the Supplementary Material. I have more comments about the experimental validation of the proposed solution:

Response: Thank you for the overview of our work and the kind assessment.

Comment 1:

As a general comment, the manuscript has a quite extensive supplementary material in terms of Appendix, while the main text is more limited, especially in terms of main Figures. I feel that some of the more important results/figures may be moved from the supplementary to the main text.

Response: Following the reviewers' suggestion, we have now moved a summary plot of the comparison experiment across the 33 microbiome datasets into the main text. Furthermore, we have now included several additional plots in the main text (see Figure 3 and 4 in the revised manuscript). If there are further experiments the reviewer would like to see in the main text, we would be happy to include them.

Comment 2:

Following the previous point, the comparison among different classifiers is summarized in Figure 3 for only a fraction of the considered datasets (8 among 33 if I understand correctly). I think it would be important to have a figure here that summarizes the results for all considered datasets.

Response: We have now replaced the previous Fig. 3 by a new figure including a summary of all 33 datasets. Fig. 3 (a) compares the median normalized score of all methods for each dataset and Fig. 3 (b) lists the times each method was significantly outperformed. In summary, these results show that KernelBiome provides a significant improvement over existing procedures. Detailed results (i.e., individual scores as well as the precision-recall curves) for the classification tasks have been moved to the appendix.

Comment 3:

Despite the extensive validation, I still don't get to which extent the proposed solution outperforms the existing ones. For example, in how many cases/datasets

the proposed method outperforms the other ones? Can you suggest (or not) your solution based on some data characteristics?

Response: See our response to Comment 2. In particular, we now perform a Wilcoxon signed rank test to check which methods are significantly outperformed by a competitor on each dataset. Importantly, KernelBiome was the method that was outperformed the least amount of times across the 33 datasets (see Fig. 3 (b)). Overall, we see KernelBiome as powerful prediction procedure that, without much manual tuning, results in state-of-the-art prediction performance across a broad range of microbiome datasets. We did not notice any particular characteristics that could be used to judge whether Kernelbiome performs good or bad. In general this will depend on the signal and whether it can be captured better by a competitor method. In the cases in which KernelBiome performed worse than one of its competitors (e.g., pcdai-rectum, pcdai-ileum, impaired-diabetes, black-hispanic, hmp-sex), KernelBiome was (almost) always able to capture useful signal (i.e., outperforms the baseline, see Fig. 3 (a) or Fig. 9 in the appendix) except for pcdai-rectum and pcdai-ileumin where none of the methods was able to outperform the baseline (indicating a very weak signal) and impaired-diabetes.

Comment 4:

Could you add a statistical test to evaluate if differences in terms of accuracies are statistically significant?

Response: We have now included a Wilcoxon signed-rank test (see our response to Comment 3 above).

Comment 5:

I find interesting that the method can deal with prior information. Which is the added value of this information in terms of classification accuracies? I think it would be relevant performing a comparison in this direction (i.e., comparing results by incorporating or not the prior information).

Response: Thank you for the suggestion. We have now added a new subsection Sec. 3.2 where we evaluate the predictive performance of our method with and without using prior information based on semi-synthetic data. Specifically, we show that the predictive performance can indeed be improved by incorporating prior information when the information aligns with the underlying data generating process (DGP), while including prior information can be harmful if it does not align with the DGP.

Comment 6:

It is not very clear to me which are the free parameters that should be set for the proposed solution. In case, a sensitivity analysis to them should be performed.

Response: One of the advantages of our framework is that we provide a full list of kernels and sane default parameters. This means that the user in practice does not need to choose any further parameters. Nevertheless, the user may want to adapt some of the parameters to either reduce the computational burden or fine tune the method. We have now clarified this in Sec. 2.3.1, where we discuss which parameters can be choosen and how this effects run time and outcome.

Comment 7:

What in terms of computational complexity of the proposed solution with respect to the compared ones? Please add some empirical evaluations.

Response: We added Fig. 3 (c) listing the average run times of each method on each of the 33 microbiome datasets. As can be seen, KernelBiome is the computationally most expensive method but it does overlap with some of the other machine learning methods. We have further added a short discussion on the theoretical run time of KernelBiome in Section 2.3.2.

Response to Reviewer #2

Summary:

In this manuscript, Huang et al., propose a kernel-based nonparametric regression and classification framework to address the challenges of compositionality and sparsity in analyzing compositional data. The authors compared the proposed framework with existing methods on publicly available microbiome datasets. Overall, I found the paper to be well-written and well-organized. I have some comments for the authors.

Response: Thank you for your kind assessment.

Comment 1:

Accuracy and MSE are applied in the classification and regression tasks, respectively. In classification tasks with unbalanced data, I don't think accuracy and AUROC are appropriate metrics. Therefore, I am highly concerned with the performance presented in the manuscript. Moreover, MSE and RMSE are

> *both commonly used metrics for evaluating the performance of regression models. MSE is in square units of the target variable, which can make it difficult to interpret the results.*

Response: We have now replaced accuracy by balanced accuracy for classification and replaced MSE by RMSE for regression. All results have been updated. We have also replaced ROC curves by precision-recall (PR) curves (Fig. 8 in Appendix). Additionally, we also updated Fig. 3 with a summary of predictive performance on all 33 datasets, and included the detailed scores in the appendix. These changes did not change the overall conclusions of the experiments and only affected some of the most unbalanced datasets.

Comment 2:

> *Line 357; Please consider add more details on the baseline.*

Response: Thanks. We have now updated the description of the baseline predictor.

Comment 3:

> *The authors claimed that "On all datasets KernelBiome achieves the best or close to best performance, indicating that the proposed procedure is well-adapted to microbiome data." However, this is not true at all when you look at Figure 6. In some case, the performance of KernelBiome is almost the worst.*

Response: We have now included a more comprehensive comparison on the 33 datasets (see Fig. 3 (a) and (b)). KernelBiome indeed does not outperform all methods on all datasets, however among all methods it was the one that was outperformed in the least amount of cases, indicating that it was the best performing method among the competitors. Moreover, in the datasets in which KernelBiome was outperformed by a competitor, KernelBiome was (almost) always able to capture useful signal (i.e., outperforms the baseline, see Fig. 3 (a) or Fig. 9 in the appendix) except for pcdai-rectum and pcdai-ileumin where none of the methods was able to outperform the baseline (indicating a very weak signal) and impaired-diabetes.
