# Supplementary material for: Supervised learning and model analysis with compositional data
Source: PLoS Comput Biol. 2023 Jun 30;19(6):e1011240. doi: 10.1371/journal.pcbi.1011240 (PMC10343141; doi:10.1371/journal.pcbi.1011240)
Supplement: Attachment — Submitted filename: point_by_point_response.pdf [file pcbi.1011240.s009.pdf]

Point-by-point response to the reviews' comments on
"Supervised Learning and Model Analysis with Compositional Data"

We thank the reviewers and the members of the editorial board for their feedback. We have addressed the remaining comment and added p-values to Fig. 3 and Fig. 4. The changes are tracked in the manuscript.

Response to members of the editorial board

> *All scientific questions have been address. We agree with reviewer #2 that adding the p-value to the figures is best practices.*

Response: Thank you for handling our manuscript. As mentioned in the summary, we have added p-values to two figures where different methods are compared.

Response to Reviewer #1

Comment 1:

> *I feel the authors have answered positively to my previous comments and modified the manuscript according to them.*

Response: Thank you again for reviewing our manuscript and the helpful feedback.

Response to Reviewer #2

Comment 1:

> *All my comments have been adequately addressed. On another note, the statistical P value should present with all the figures when comparing different methods. By including P-values, readers can better assess the significance of the findings and make informed interpretations.*

Response: Thank you again for the helpful feedback. We have now added p-values to all figures comparing multiple methods.
